# Therapeutic targets and biomarkers of tumor immunotherapy: response versus non-response

**DOI:** 10.1038/s41392-022-01136-2

**Published:** 2022-09-19

**Authors:** Dong-Rui Wang, Xian-Lin Wu, Ying-Li Sun

**Affiliations:** 1 Bone Marrow Transplantation Center, The First Affiliated Hospital, School of Medicine, Zhejiang University, Hangzhou, China; 2 Liangzhu Laboratory, Zhejiang University Medical Center, Hangzhou, China; 3 Institute of Hematology, Zhejiang University, Hangzhou, China; 4 Zhejiang Province Engineering Laboratory for Stem Cell and Immunity Therapy, Hangzhou, China; 5 Central Laboratory, National Cancer Center/National Clinical Research Center for Cancer/Cancer Hospital & Shenzhen Hospital, Chinese Academic of Medical Sciences and Peking Union Medical College, Shenzhen, China; 6 CAS Key Laboratory of Genomic Precision Medicine, Beijing Institute of Genomics, Chinese Academy of Sciences, Beijing, China

**Keywords:** Immunotherapy, Cancer

## Abstract

Cancers are highly complex diseases that are characterized by not only the overgrowth of malignant cells but also an altered immune response. The inhibition and reprogramming of the immune system play critical roles in tumor initiation and progression. Immunotherapy aims to reactivate antitumor immune cells and overcome the immune escape mechanisms of tumors. Represented by immune checkpoint blockade and adoptive cell transfer, tumor immunotherapy has seen tremendous success in the clinic, with the capability to induce long-term regression of some tumors that are refractory to all other treatments. Among them, immune checkpoint blocking therapy, represented by PD-1/PD-L1 inhibitors (nivolumab) and CTLA-4 inhibitors (ipilimumab), has shown encouraging therapeutic effects in the treatment of various malignant tumors, such as non-small cell lung cancer (NSCLC) and melanoma. In addition, with the advent of CAR-T, CAR-M and other novel immunotherapy methods, immunotherapy has entered a new era. At present, evidence indicates that the combination of multiple immunotherapy methods may be one way to improve the therapeutic effect. However, the overall clinical response rate of tumor immunotherapy still needs improvement, which warrants the development of novel therapeutic designs as well as the discovery of biomarkers that can guide the prescription of these agents. Learning from the past success and failure of both clinical and basic research is critical for the rational design of studies in the future. In this article, we describe the efforts to manipulate the immune system against cancer and discuss different targets and cell types that can be exploited to promote the antitumor immune response.

## Introduction: The history of tumor immunotherapy

In 1891, William B Coley, an orthopedic surgeon at New York Memorial Hospital in the United States, injected bacteria into tumors to treat cancer.^1,2^ There were few developments in the use of tumor immunotherapy until specific immune cells and immune-regulating molecules were identified. In 1974, interleukin (IL)-2 was discovered to play an essential role in T-cell differentiation and growth, and its utilization on cancer patients by Steven Rosenberg and his team was a milestone of tumor immunotherapy in the modern era,^3–5^ which also led to many approaches in the 1980s involving the application of cytokines for stimulating immune responses in patients with cancer.^6,7^ However, direct application of cytokines to patients can result in significant side effects,^8–10^ which warrants the discovery of specific immune cells that mediate the antitumor response and can be precisely targeted.

Activation of T cells is a key event in both antiviral and antitumor adaptive immunity, which is mainly accomplished through dual signaling pathways. The first signal is an antigen-specific signal, which involves the specific binding of the T-cell surface receptor (TCR) to the antigenic peptide-major histocompatibility complex (MHC).^11,12^ The second signal is mediated by the communication of T cells with costimulatory molecules (CMs) on the surface of antigen-presenting cells (APCs).^13^ These “primed” T cells can produce perforin and granzyme, which lyse target cells, and can secrete cytokines and induce target cell apoptosis through the combination Fas-FasL interaction.^14^ Blocking the activation of T cells against malignant cells in cancer patients has been the central problem for tumor immunology research.^15–17^

After the identification of T-cell receptors (TCRs) that are responsible for antigen recognition,^18,19^ in 1986, scientists discovered the molecule CD28 expressed on activated T cells.^20^ Subsequently, it was found that T-cell activation requires both signals from the TCR and CD28, and CD28 was thereafter named a “costimulatory molecule”.^21–23^ Around the same time, Pierre Golstain’s team discovered a protein with a similar structure to CD28; it was named cytotoxic T lymphocyte-associated antigen 4 (CTLA-4)^24^ and hypothesized to be a potential T-cell activating molecule.^25,26^ The concept that CTLA-4 is a positive immune regulator was also shown in other studies^22^ but was later challenged by the teams of James Allison and Jeffery Bluestone, who independently discovered that blocking CTLA-4 enhanced the T-cell immune response.^27,28^ Consistently, disrupting the CTLA-4 gene was lethal in mice due to excessive immune activation, supporting the immunosuppressive function of CTLA-4.^29,30^ This discovery paved the way for Allison’s team to test whether blocking CTLA-4 can potentiate antitumor immunity and inhibit the growth of immunosuppressive tumors. At the end of 1994, Allison’s team developed an antagonistic CTLA-4 antibody to be evaluated in tumor-bearing mice and later reported the ground-breaking discovery that blocking CTLA-4 can increase the antitumor activity of T cells and inhibit tumor growth.^31^ Therefore, for the first time, it was demonstrated that inhibiting a negative immune regulator could suppress tumor progression; this approach was later named “immune checkpoint blockade” (ICB) by Allison.^32^ In 1997, Allison’s team suggested that both inducing T-cell costimulatory signals and reducing inhibitory signals can be potential approaches for cancer immunotherapy.^33^ In 2011, ipilimumab, the first antibody targeting CTLA-4, was approved for melanoma treatment and became the first immune checkpoint (IC) inhibitor.^34,35^

More than 20 years ago, the research group of Tasuku Honjo at Kyoto University discovered programmed cell death protein 1 (PD-1).^36^ PD-1 knockout led to autoimmune disease and abnormally activated immune cells in mice,^37^ suggesting its immune-suppressive role. In 1999, the research group of Lieping Chen at the Mayo Clinic discovered a molecule named B7-H1,^38^ which was later found to be expressed on tumor tissues such as melanoma and lung cancer and can promote the apoptosis of tumor-specific T cells, making them unable to attack cancer cells.^39^ In 2000, B7-H1 was identified as a ligand of PD-1, therefore acquiring its second name PD-L1.^40^ In 2002, PD-L2 was discovered, and the signaling pathway involving PD-1 was clarified.^41,42^ These discoveries demonstrated that PD-1 is another IC. Indeed, PD-1/L1 inhibitors are the most widely applied immunotherapy type to date, with 6 drugs that have been approved in the United States. In China, 4 PD-1 inhibitors have been approved for commercialization.^43^ The approved PD-1 inhibitors and PD-L1 inhibitors have changed the paradigm of cancer therapy.^44–47^

The concept of cellular immunotherapy arose from the observation of graft-versus-leukemia effects in allogeneic bone marrow transplantation.^48^ To support in vivo maintenance and tumor recognition, cell engineering technologies were integrated with adoptive transfer. The first chimeric antigen receptor (CAR) was generated in 1989 by the Zelig Eshhar group in Israel.^49^ These “first-generation” CARs are the variable regions of antibodies fused to the TCR signaling domain, which mediates T-cell activation against the targeted antigen but has shown limited in vivo expansion. In 2002, the Michel Sadelain group incorporated the costimulatory domain into the CAR construct.^50^ The resulting “second-generation” CARs have seen extraordinary clinical responses against malignancies of the B-cell lineages, including B-cell chronic lymphocytic leukemia (B-CLL) and B-cell acute lymphocytic leukemia (B-ALL).^51^ On 30 August 2017, the US FDA approved the first CAR-T product, Kymriah from Novartis, for the treatment of relapsed or refractory patients under the age of 25 with acute B-cell lymphoblastic leukemia (B-ALL). The commercialization of CAR-T-cell therapy soon followed with the approval of six other products targeting leukemia, lymphoma, and multiple myeloma.^52^ At present, ~1000 registered clinical studies are ongoing to evaluate CAR-T-cell immunotherapy against leukemia, lymphoma, melanoma, glioma and other malignant tumors.^53–59^

**Fig. 1:**
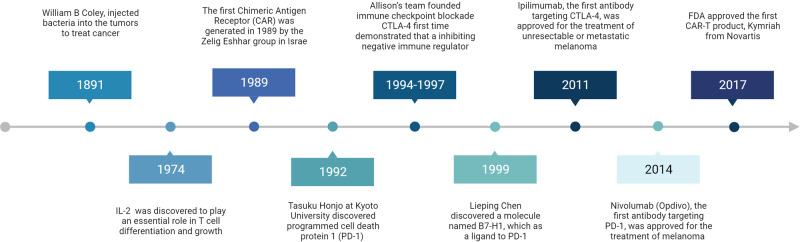
Historical landmarks in cancer immunotherapy development

We summarized the major milestone breakthroughs in cancer immunotherapy over time (Fig. 1).

Antibodies against CTLA-4 and PD-1, as well as CAR-T-cell therapy, represent promising approaches in which certain components of the immune system can be manipulated to reverse suppression and target tumors. However, not all patients respond to these therapies, indicating the complexity of tumor-induced immune alteration. In 2006, the concept of “cancer immunoediting” was introduced by Dr. Robert Schreiber, describing how malignant cells can respond to initial immune recognition and subsequently develop escape mechanisms and even “reprogram” the immune system to become protumorigenic.^60,61^ Such an immunoediting process can occur almost every time intratumoral or systemic immune cells are present, resulting in a highly suppressive tumor microenvironment (TME). The search for a synergistic approach for activating antitumor T-cell responses and targeting the suppressive TME has been the main focus of research in tumor immunotherapy.

New targets and drug candidates have been emerging for cancer immunotherapy, but most are still in the very early stage of development. Unfortunately, clinical studies have revealed that quite a few of these candidates may not exert satisfactory outcomes as monotherapies. In this article, we will discuss different strategies for cancer immunotherapy, including IC- and stimulatory molecule-targeted agents, cellular immunotherapy, and suppressive TME-targeting strategies. Furthermore, as ICB and CAR-T cells have been the most rigorously evaluated immunotherapy strategies in the clinic, we will also discuss biomarkers associated with the clinical efficacy of these two types of treatment.

## ICs on T cells

**Fig. 2:**
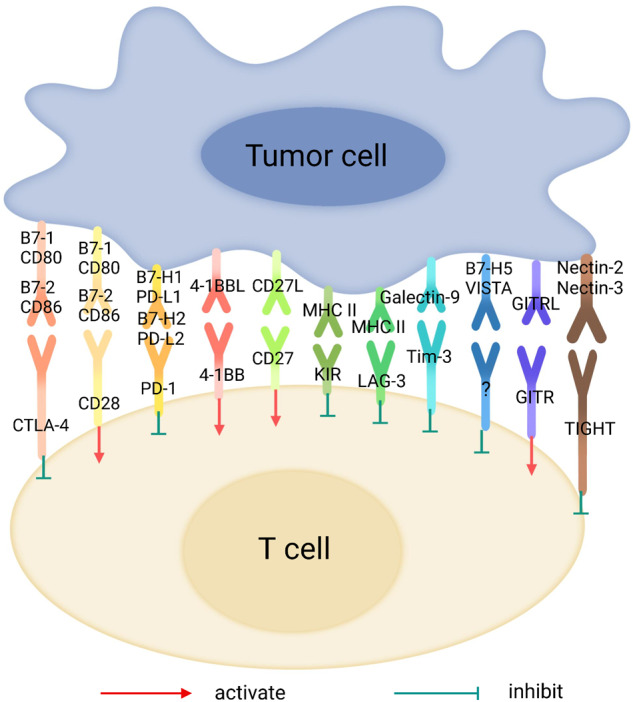
List of immune checkpoint inhibitors and their receptors

ICB has revolutionized the field of cancer therapy and has become one of the most valuable methods in the treatment of many late-stage cancers.^62–64^ ICs are a class of immunosuppressive molecules that are expressed on immune cells and can suppress immune cell activation, therefore playing a key role in autoimmunity prevention (Fig. 2).^65–68^ In contrast, overexpression of ICs suppresses immune function and contributes to tumorigenesis.^69–73^ ICB therapy, therefore, inhibits tumor growth by blocking ICs and potentiating antitumor T-cell activity.^74–77^ The development of ICB was initiated by targeting two IC pathways, PD-1/PD-L1 and CTLA-4/B7-1/2,^78–80^ the blocking of which has made remarkable clinical progress, especially against non-small cell lung cancer, colon cancer, melanoma, and renal cell carcinoma.^81–88^ However, only 20–30% of patients achieve long-term survival following these ICB treatments, and one of the underlying mechanisms is the expression of other inhibitory molecules.^88^ Therefore, the continuous identification of new IC targets and the development of their corresponding ICBs have become critical. To date, several other IC molecules on T cells that mediate inhibitory signals through different mechanisms have been identified, with the potential to be exploited as targets for cancer immunotherapy.

One of the key processes involved in cancer development is that cancer cells acquire immune escape by inducing and recruiting immunosuppressive cells, such as Treg cells, bone marrow-derived suppressor cells, and tumor-associated macrophages, as well as increasing the expression of various immunosuppressive molecules, such as PD-1 and PD-L1 (PD-L1). Blocking these immunosuppressive mechanisms can restore the underlying antitumor immune response. Cancer immunotherapy requires ICB, such as CTLA-4 and monoclonal antibodies against PD-1 or PD-L1, which restore the function of cytotoxic effector CD8^+^ T cells and kill cancer cells, leading to tumor suppression and a paradigm shift in cancer treatment for many cancer types. However, since more than half of treated patients do not respond to ICB even in combination with other therapies, the identification of biomarkers that predict clinical efficacy is an urgent issue.^89^

### PD-1

PD-1 was found to inhibit the function of T lymphocytes, which is critical in controlling the autoimmune response.^90–94^ PD-L1 (initially identified as B7-H1), is highly expressed on multiple types of tumors and can bind to PD-1 and mediate tumor immune escape.^39^ Therefore, inhibition of PD-1 can reactivate T-cell function.^95,96^ Recent studies have also revealed that PD-1 is expressed not only on T cells but also on NK cells, B lymphocytes, macrophages and dendritic cells (DCs),^97,98^ suggesting that PD-1 may play a very effective role in remodeling the tumor immune microenvironment and even systemic antitumor immunity.^46,99–102^

**Fig. 3:**
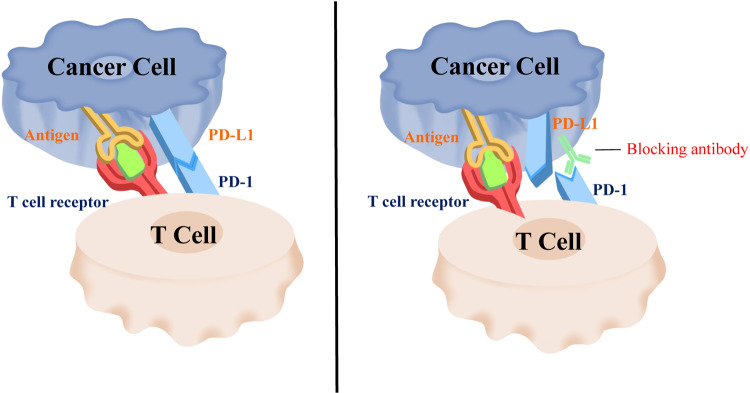
Schematic diagram of the working mechanism of PD-1 antibodies

PD-1 inhibitors can specifically bind to PD-1, thereby attenuating the immunosuppressive regulation of T lymphocytes and enabling T lymphocytes to participate in the killing of tumor cells (Fig. 3).^84^ Preclinical studies have shown that PD-1 inhibitors can inhibit the proliferation of cells and induce the programmed cell death (apoptosis) of various tumor cells.^103^ PD-1 antibodies can also enhance the apoptosis of tumor cells mediated by other cytotoxic agents, such as adriamycin.^104,105^

The clinical efficacy of PD-1/L1 blocking antibodies was first observed against tumors with high PD-L1 expression, including melanoma, non-small cell lung cancer (NSCLC), and renal cell carcinoma (RCC).^42,106,107^ The PD-1 blocking antibody nivolumab (Opdivo) was approved in 2015 for advanced squamous cell lung cancer treatment, marking the first clinical application of anti-PD-1 therapy. Of note, the prescription criteria did not include the expression of PD-L1 on tumor cells.^108,109^ After that, pembrolizumab was the first immunotherapeutic drug approved by the FDA for first-line treatment of patients with metastatic NSCLC in 2016.^110–112^ Different from nivolumab, the prescription of pembrolizumab requires confirmed PD-L1 overexpression on tumors.^113,114^ Nivolumab and pembrolizumab (coreda) were later approved as single agents for the second-line treatment of NSCLC (non-small cell lung cancer).^108,115–117^ On the other hand, atezolizumab (Tecentriq) was approved in 2016 to treat patients with metastatic NSCLC and disease progression during or after first-line platinum chemotherapy.^117,118^ In addition, atezolizumab can also be used in patients with EGFR mutations or ALK rearrangements undergoing targeted therapy and disease progression.^119–121^ Another two new PD-L1 antibodies, durvalumab (Imfinzi) and avelumab (Bavencio), were approved in 2017.^122,123^ Furthermore, sindilizumab, a PD-1 antibody developed by Innovent Biologics in China, also achieved good results after two cycles of neoadjuvant administration,^124^ representing another candidate to target this pathway.^125^

However, more than 50% of patients with cancer do not respond to PD-1/L1 inhibitors. Of note, the objective response rate was only 45% with pembrolizumab for non-small cell lung cancer, even in patients with high expression of PD-L1.^126^ In addition, a small proportion of patients experience hyperprogressive disease (HPD),^127–130^ which may be a result of regulatory T-cell (Treg) outgrowth and subsequent inhibition of antitumor immunity.^30,131–133^ These results suggest that PD-1 blockade needs to be prescribed in a personalized manner to maximize its efficacy.

### CTLA-4

CTLA-4, also known as CD152, is a transmembrane protein expressed in activated CD4^+^ and CD8^+^ T cells.^134,135–138^ While CD28 was found to be a T-cell costimulatory molecule,^139^ CTLA-4 was later discovered to mimic CD28 and act as a brake on T-cell activation.^140,141^ Under physiological conditions, CTLA-4 and CD80/CD86 binding can inhibit T-cell activation signals and prevent autoimmune disease.^142,143^ Blocking CTLA-4 can directly target inhibitory signals on effector T cells and reduce the inhibitory effect of Tregs,^33,144–147^ thus effectively enhancing the antitumor effect of T cells.

In 1996, James Allison found that blocking CTLA-4 caused tumor regression in mice.^148^ In subsequent human studies, the CTLA-4 antibody ipilimumab was the first-in-class ICB agent to be tested in clinical studies. Ipilimumab performed well and successfully inhibited disease progression in patients with refractory metastatic melanoma, which was a milestone of cancer immunotherapy.^149^ Intriguingly, CTLA-4 was particularly highly expressed on the surface of Treg cells infiltrated by melanoma, lung cancer and kidney cancer.^150^ Although it was later found that Treg depletion is not the main mechanism of the clinical antitumor efficacy of ipilimumab,^144^ these results suggest that the CTLA-4 antibody may also inhibit Treg cells in the TME under certain circumstances and contribute to immune activation.^136,151,152^

**Fig. 4:**
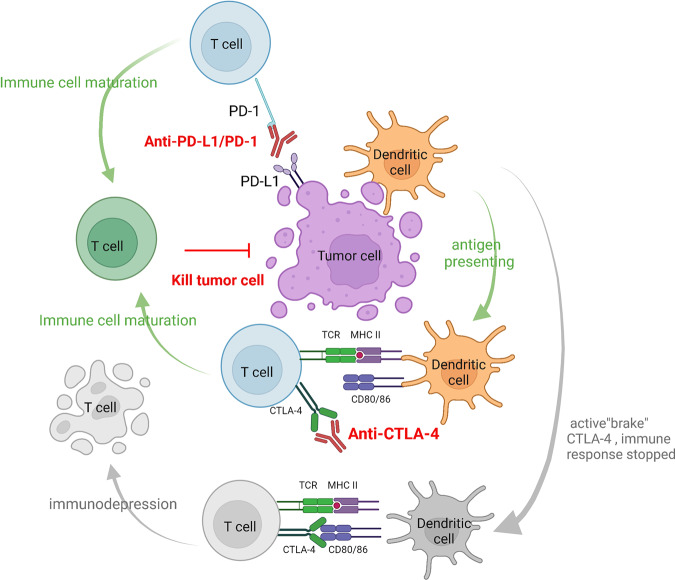
Illustration of CTLA-4 and PD-1

Although both are representative IC molecules, CTLA-4 and PD-1 regulate T-cell function in different manners (Fig. 4). While the inhibitory signal from CTLA-4 negatively regulates T-cell priming, PD-1 mainly mediates the subsequent activation and proliferation of primed T cells.^153^ In the context of tumors, it was found that ICB targeting PD-1 usually leads to the expansion and recruitment of existing antitumor T cells, while anti-CTLA-4 therapy generates new T-cell clones.^153,154^ Furthermore, anti-CTLA-4 therapy was found to induce a Th1-like CD4^+^ subset, which was not observed in anti-PD-1 therapy.^155^ Genetic models also revealed that CTLA-4 enforces boundaries on CD4^+^ T-cell phenotypes and that PD-1 subtly restrains CD8^+^ T-cell phenotypes.^156^ These results indicate that CTLA-4 and PD-1 may be simultaneously targeted for synergistic antitumor effects. The combinational therapy of CTLA-4 and PD-1 ICBs indeed resulted in superior clinical responses but led to more significant adverse effects than monotherapy.^153^

### Tim-3

T-cell immunoglobulin domain and mucin domain-3 (Tim-3, CD366) is a T-cell surface inhibitory molecule that is mainly expressed on CD4^+^ T helper cell 1 (Th1) and CD8^+^ CTL cells^157–160^ and on a subset of Treg cells with enhanced inhibitory function.^161,162^ Tim-3, also known as HAVCR2, was later found to also be expressed on some innate immune cells, including dendritic cells, NK cells, monocytes, and macrophages.^163^ In IA/IB studies, the Tim-3 blocking antibody LY3321367 was well tolerated as a single agent or in combination with an anti-PD-L1 antibody.^164^ In addition, in one patient with extensive stage PD-L1-negative small cell lung cancer that was resistant to cisplatin/etoposide and PD-1/CTLA-4 antibodies, anti-TIM-3 monotherapy resulted in a partial response (PR). Therefore, preliminary antitumor activity of anti-TIM-3 therapy was observed in early clinical studies, but phase II and III studies are still needed to verify the efficacy in larger cohorts of patients.^165^

### LAG-3

LAG-3 can be induced on CD4^+^ and CD8^+^ T cells under antigen stimulation. The inhibitory function of LAG-3 is closely related to its expression level on the cell surface, which is under stringent regulation during homeostasis.^166–168^ Long-term infection with viruses, bacteria and parasites causes continuous exposure to antigens, which leads to high levels and continuous expression of LAG-3 and subsequent reductions in cytokine release, cytolytic activity, and proliferation potential.^169–172^ Coexpression of LAG-3 and PD-1 on intratumor T cells has been observed in several mouse tumor models, and synergistic inhibition of tumor growth was observed when combining the blocking antibodies of these two molecules.^173–176^

LAG-3 has thus become one of the most critical new targets of cancer immunotherapy and is considered a major development direction after PD-1 with great application prospects.^171,177,178^ Relatlimab, the first inhibitor of LAG-3 to enter the clinic, blocks the interaction of LAG-3 with MHC II.^179^ RELATIVITY 047 (CA224-047), a phase II/III clinical study, was designed to evaluate a fixed-dose combination of relatlimab combined with nivolumab versus nivolumab monotherapy in patients with previously untreated metastatic or unresectable melanoma. The study resulted in a median progression-free survival (PFS) of 10.12 months (95% CI, 6.37– 15.74) in the combination group compared with 4.63 months (95% CI, 3.38–5.62) in the monotherapy group. In addition, the PFS rates at 12 months were 47.7% and 36.0%, respectively, supporting further development of anti-LAG-3 treatment.^180^

In 2019, Wang et al. identified fibrinogen-like protein 1 (FGL1) as the ligand for Lag-3.^181^ It was found to bind to Lag-3 to form a new PD-1/PD-L1-independent immune checkpoint pathway, leading to T-cell exhaustion, dysfunction, and tumor cell evasion of immune surveillance. Blocking FGL1 in addition to anti-PD-L1 has the potential to become another novel ICB strategy in clinical practice, especially in the targeted therapy of non-small cell lung cancer (NSCLC).^182^

### NR2F6

Nuclear receptor subfamily 2 group F member 6 (NR2F6) was recently reported as an intracellular IC molecule, which is an orphan nuclear receptor inherent to lymphocytes.^183,184^ NR2F6 acts as a transcription factor regulating the activation, recruitment, proliferation, and homeostasis of cells associated with tumor antigen-specific T-cell responses. In NSCLC tissues, high expression of NR2F6 was detected in tumor-infiltrating lymphocytes (TILs), and upregulated NR2F6 expression was associated with impaired production of cytokines, including IL-2, TNF-α, and IFN-γ,^185^ suggesting that NR2F6 on TILs contributes to tumor immunosuppression. Moreover, the disruption of NR2F6 resulted in tumor suppression and enhanced the effect of PD-L1 blockade in tumor therapy, suggesting that NR2F6 inhibitors may become a new type of immunotherapy that can overcome resistance to existing ICB treatment.^186,187^

### TIGIT

T-cell immunoglobulin and ITIM domain protein (TIGIT) is a type I transmembrane protein. TIGIT belongs to the immunoglobulin superfamily (IgSF) and can be expressed on T cells, regulatory T cells, memory T cells, and NK cells. TIGIT mediates the inhibitory effect on the activation of NK cells and T cells through its interaction with the ligands CD155 and CD112, which are expressed on antigen-presenting cells (APCs).^188–191^ In human tumors, TIGIT was found to be coexpressed with multiple IC molecules, including PD-1, TIM-3, and LAG-3.^192^ The coexpression of TIGIT, TIM-3 and PD-1 showed a correlation with poor survival in patients.^193^ In mouse models of malignant melanomas, it was found that the tumor growth rate was slowed down after TIGIT knockout, and survival was significantly prolonged.^194^ In human cancer models, simultaneous blockade of the TIGIT and PD-1 signaling pathways increased the expression of IFN-γ and TNF-α in tumor-specific CD8^+^ T cells, supporting the development of anti-TIGIT treatment. Two phase I clinical studies targeting TIGIT for cancer immunotherapy are currently ongoing.^193^

### VISTA

V-set immunoregulatory receptor (VISTA), also known as PD-1H or DD1α, is an immunomodulatory protein that was discovered in recent years. It is mainly expressed in lymphoid organs and bone marrow cells, and its structure is similar to that of PD-L1.^195,196^ Studies have shown that VISTA-expressing APCs have inhibitory effects on CD4^+^ and CD8^+^ T cells; when this molecule is blocked, the immune function mediated by T cells is rescued, suggesting that VISTA is an IC molecule that inhibits T-cell responses.^196^ In T cells, the inhibitory effects of VISTA and PD-1 are independent of each other, and studies in mouse models of tumors also verified that the simultaneous application of anti-PD-1 and anti-VISTA antibodies can inhibit tumor growth and prolong survival.^197^

In gastric cancer patients, VISTA was found to be expressed in some tumor cells, as well as TILs. Furthermore, patients with VISTA-high oral squamous cell carcinoma have a poor prognosis. After treatment with ipilimumab in prostate cancer patients, the levels of VISTA^+^ TILs and macrophages were significantly upregulated, indicating that VISTA might contribute to acquired resistance to current ICB treatments, and the combined blockade of VISTA and CTLA-4 may exert better effects than blockade of either factor alone. The anti-VISTA antibody JNJ-61610588 is now being evaluated in a phase I clinical study for the treatment of solid tumors (NCT02671955).^198^

### BTLA

B and T lymphocyte attenuator (BTLA) belongs to the immunoglobulin superfamily. It is expressed on T cells, resting B cells, macrophages, dendritic cells and NK cells and is similar in structure and function to PD-1 and CTLA-4. The ligand for BTLA is herpesvirus entry mediator (HVEM). When BTLA binds to HVEM, it generates inhibitory signals and inhibits T-cell activation. Anti-BTLA treatment can promote T-cell proliferation, and BTLA knockout mice show higher immune activity.^199–201^ In patients with malignant melanoma, tumor-specific T cells in both circulating lymph and metastatic lymph nodes expressed BTLA, and the expression of HVEM was also detected in the patient’s tumor tissue.^202^ The expression of BTLA was found to be significantly increased in pleural effusion samples from patients with lung cancer, which is an indicator of tumor aggressiveness.^203^ Therefore, BTLA, as an inhibitory molecule for immune regulation, has broad research prospects. At present, research on BTLA and HVEM inhibitors is still in the preclinical stage, and it is expected that related drugs will be launched as soon as possible and enter into clinical research verification.^204–206^

## Immune stimulatory molecules on T cells

### OX40

OX40, also known as CD134, is a member of the tumor necrosis factor receptor (TNFR) superfamily and is expressed 24–72 h after T-cell activation. Its ligand OX40L, also known as CD252, is mainly expressed on the surface of activated APCs. The OX40-OX40L interaction can initiate T-cell activation signals as well as the expression of cyclin A, Bcl-2 anti-apoptotic molecules, cytokines, and cytokine receptors.^207^ Mouse models have shown that specific antibodies that stimulate OX40 can reduce the number of Tregs, thereby maintaining the function of effector T cells and showing high antitumor activity.^208–210^

There are many clinical studies targeting the OX40-OX40L pathway, including those on single-agent application of specific antibodies that excite OX40 or combination with chemotherapy, radiotherapy, surgery, small molecule-targeted therapy, cytokines or other ICB drugs.^211^ The results of a study of the OX40 agonist MOXR0916 showed that as a monotherapy or in combination with atezolizumab, the treatment achieved PR in 2 out of 51 patients, and more phase I/II clinical studies are underway. However, there have been no clues about whether OX40 agonists should be used as a monotherapy or combined with other drugs. Future basic and clinical research is needed for an in-depth understanding of the mechanism by which OX40 regulates different T-cell subtypes in the TME.

### ICOS

Inducible costimulatory molecule (ICOS), also known as CD278, is a member of the immunoglobulin superfamily. ICOS is expressed on the surface of activated T cells and regulates T-cell proliferation and function.^212^ The activation of ICOS is dependent on its ligand ICOS-L, which is mainly expressed in B cells and APCs. ICOS has been shown to be an important marker for ICB efficacy.^212–214^ When malignant melanomas were treated with anti-CLTA-4, the abundance of ICOS^+^ CD4^+^ T cells was found to be associated with better efficacy.^155^ In mouse models, ICOS agonist alone had difficulty eliciting sufficient antitumor responses against melanoma; however, there was a synergistic effect between ICOS and anti-CTLA-4; in addition, ICOS knockout mice responded poorly to anti-CTLA-4 treatment.^215^ Simultaneous use of ICOS agonists and anti-PD-1 and anti-CTLA-4 therapy can also enhance the antitumor effect against lung cancer in preclinical models.^216^ Currently, several phase I clinical studies targeting the ICOS pathway are ongoing.^217^

### 4-1BB

4-1BB, also known as CD137, is a member of the TNFR family.^218^ The function of 4-1BB on regulatory T cells is complex, and studies have led to contradictory results. However, importantly, 4-1BB gene knockout mice developed autoimmune diseases, suggesting that it plays an important role in immune balance and has the potential to be targeted to elicit tumor-specific immune recognition.^219,220^

Currently, clinical studies of two 4-1BB-specific agonistic antibodies, urelumab and PF-05082566, are ongoing, and the preliminary results support that 4-1BB agonism can promote the proliferation and activity of T cells and NK cells.^221^ Another clinical study used PF-05082566 combined with PD-1 antibody to treat NSCLC and renal cell carcinoma. With only 6 out of 23 patients achieving a complete response (CR) or PR, combinational therapy may need to be reinvestigated for better patient selection.^219^

### CD27

Unlike other members of the TNFR family, CD27 is expressed only on the surface of lymphocytes, including naive and activated CD4^+^ and CD8^+^ T cells. When it interacts with its ligand CD70, CD27 induces the proliferation and differentiation of effector and memory T cells and enhances the activation of B cells and NK cells.^222,223^ Mouse models suggest that the induction of the CD27 signaling pathway can inhibit tumor growth.^224^ In addition, it was found that 1/3 of patients with Hodgkin's lymphoma and diffuse large B-cell lymphoma had germline depletion of CD27 or CD70, and most patients with diffuse large B-cell lymphoma and Burkitt's lymphoma had mutation or depletion of the CD70 gene, which further verified the antitumor immune effect of the CD27/CD70 signaling pathway.^225–228^

Varlilumab is a humanized monoclonal antibody against CD27 that promotes cytokine production and activation of T cells. In a phase I clinical study, varlilumab was well tolerated in patients with advanced solid tumors and has shown initial safety results: of 56 patients in the phase I study, only one patient developed grade 3 hyponatremia, and most treatment-related toxicities were grade 1–2.^229^ One patient with advanced renal cell carcinoma achieved a PR (tumor reduction of 78%), which lasted for 2.3 years. Eight patients had stable disease (SD) for more than 3 months, and one patient with advanced renal cell carcinoma had SD for more than 3.9 years.^229^ In addition to monotherapy, varlilumab has also been used in combination with anti-PD-L1 antibodies.^230^ The antitumor effect of this CD27 agonist still needs further, larger-scale investigation.^227^

### ICs on NK cells

In the human body, NK cells are mainly characterized by a CD3-CD56^+^ lymphocyte population, and the CD16^+^CD56dim subtype is mainly found in the blood. As an important part of the natural immune system, NK cells play an important role in removing senescent cells and pathogenic microorganisms.^231^ NK cells do not recognize target cells through specific receptors like TCRs do; they recognize cells through receptors expressed by germline genes. The negative regulators of NK cells include KIRs (immunoglobulin-like receptors), CD94-NKG2 and MHC-I.^232–234^ In the context of tumor immunology, as tumor cells downregulate MHC expression to escape acquired immunity, they become more susceptible to NK-cell cytotoxicity. In addition, NK cells play an essential role in mediating antibody-induced cellular cytotoxicity (ADCC) in antibody therapy.^235–237^ NK cells can also directly exert an antitumor effect by secreting cytokines or mobilizing immune cells such as dendritic cells, macrophages, and T cells to participate in the process of removing tumor cells,^238^ making them attractive targets for cancer immunotherapy.

### KIR

The killer cell immunoglobulin-like receptor (KIR) family is a class of highly polymorphic molecules mainly expressed on the surface of some NK cells and T cells, which can be divided into multiple subtypes. Among them, KIR2DTl1-3 and KIR3DL1 can exert inhibitory effects by binding MHC molecules (HLA-C/HLAB).^239^ Due to the characteristics of high gene polymorphism, the combination of multiple KIR genes and their ligands can cause a variety of diseases, including autoimmune diseases, especially the combination of some KIR genes and specific ligands, which can increase the risk of cancer.^240^ In mouse models, treatment targeting the activated NK-cell surface receptor KIR2DS2 showed significantly superior antitumor activity to treatment targeting conventional costimulatory molecules.^241–243^

The KIR inhibitor IPH2101 showed efficacy in preclinical models but not in phase I/II clinical studies,^244^ despite the observation that KIR inactivation was associated with prolonged survival in patients with colorectal cancer and glioblastoma.^245,246^ By further analyzing the blood sample of patients treated with IPH2101, Carlsten et al. found that IPH2101 binding to KIR resulted in NK-cell clearance via FcγR recognition from APCs.^247^ Lirilumab, another inhibitor of KIR, had an objective response rate of 24% in combination with nivolumab in advanced head and neck tumors, which may suggest less APC-mediated clearance using this antibody. A phase I/II clinical study of lirilumab is underway for advanced solid tumors and hematological tumors.

### NKG2A

NK-cell lectin-like receptor subfamily C member 1 (NKG2A) is an “inhibitory” member of the NKG2 family and is mainly expressed in CD56^hi^ NK cells, NKT cells and CD8^+^αβ T-cell subsets.^248^ It forms a heterodimeric receptor with CD94 and binds with its ligand, the nonclassical MHC I molecule HLA-E, which is expressed in most normal tissues. The interaction between NKG2A/CD94 and HLA-E can inhibit the activation of NK cells and T cells,^249^ indicating the potential to be targeted as an IC molecule.

Monalizumab (IPH2201), jointly developed by Innate Pharma and AstraZeneca, is an NKG2A monoclonal antagonistic antibody that can block the interaction between NKG2A and HLA-E and has shown therapeutic effects in a leukemia mouse model.^250^ Monalizumab has been used in phase II clinical trials against cancers of the female reproductive system and NSCLC.^251^ In these clinical studies, monalizumab was well tolerated but with limited therapeutic effect, and only showed short-term SD in some patients. However, monalizumab combined with cetuximab (EGFR blocking antibody) reached a 27.5% response rate against recurrent or metastatic head and neck squamous cell carcinoma, suggesting that combining monalizumab with targeting oncogenic pathways may enhance clinical antitumor efficacy.^251^

### CD96

CD96 is a member of the immunoglobulin superfamily expressed on NK cells, recognizing the ligand CD155. It was found that CD96 expression on tumor-infiltrating NK cells was higher than that on NK cells in surrounding tissue.^252^ Higher levels of CD96 expression on NK cells in hepatocellular carcinoma samples predicted poor prognosis.^253^ Preclinical results in tumor models with implantable or spontaneous metastases have proven that CD96 inhibitors can reduce metastatic potential,^187,254^ which supports the future clinical application of agents with this target.

## Cellular immunotherapy

Immune cells with cytotoxic potential, including T cells, NK cells and macrophages, recognize and eliminate infected or damaged cells under physiological conditions. The cytotoxic effect from T cells is distinct from others given its nature of antigen specificity. Cellular immunotherapy, also called adoptive cell transfer (ACT), exploits the killing capability of these types of immune cells for the treatment of cancers.^255–258^ Here, we discuss four major types of ACT that have achieved significant research and clinical progress: CAR T-cell therapy, TIL therapy, engineered TCR therapy, and NK cell therapy.

### TIL therapy

TILs are heterogeneous lymphocytes that can be identified and purified from tumor tissues, and their abundance has been found to correlate with a better prognosis.^259–261^ Unfortunately, in most cancer patients, there are too few endogenous TILs to elicit a sufficient antitumor response. TILs were among the first set of cells exploited for ACT. These cells can be isolated from tumors, expanded in a laboratory environment in vitro, and reinjected in large numbers into cancer patients to eliminate tumor cells.^262–264^ TIL therapy has been tested rigorously in clinical studies, resulting in inspiring outcomes against certain types of tumors, with the longest reported survival of 11 years.^265,266^ TIL therapy has resulted in clinical remissions in some patients who have exhausted all other treatment options. One of the examples was a patient named Melinda Bachini, who was diagnosed with cholangiocarcinoma in 2009 and developed whole-body metastasis despite surgery and chemotherapy. This patient was then recruited into a clinical study of TILs. Only 1 month after TIL treatment, her whole-body tumor began to regress, and her physical strength recovered quickly. Now she is the first survivor of advanced bile duct cancer for more than 10 years. Another patient had metastatic adenocarcinoma that did not respond to chemoradiation and had metastases to retroperitoneal lymph nodes and to the surface of the liver. Before TIL treatment, tumor metastases were found in the retroperitoneum, abdominal wall, parahepatic and pelvic cavity. After treatment, a CR was declared in this patient with the regression of tumor sites detected.^267^ TILs have been considered to have the ability to accurately identify tumor antigens, which contributes to their tumor specificity, and this new therapy can be considered “tailored” to the patient.^268^ Below, we summarize the recent progress of TIL therapy against different types of tumors.

#### Melanoma

LN-144 (Lifileucel) is a TIL-based therapy against melanoma. A phase II clinical trial showed disease control rate of 80.3% and an objective response rate of 36.4%: two patients had a CR, 23 had a PR, and some patients' tumors completely disappeared after 2 years of treatment.^268^ More strikingly, some patients with PD-L1-negative tumors, who were likely not responsive to anti-PD-1 ICB, also responded to TIL therapy, suggesting that patients refractory to other types of immunotherapy can still benefit from TIL therapy. Indeed, another phase I clinical study used TILs to treat anti-PD-1-resistant tumors: two patients achieved a CR that lasted for more than 1.5 years,^269,270^ suggesting that for patients who have progressed after PD-1 therapy, TIL therapy is among the few other treatment options.

#### Lung cancer

A phase I clinical trial result was announced at the 2021 AACR meeting. In 12 evaluable patients with NSCLC, TIL therapy achieved a 25% overall remission rate. At a mean follow-up of 1.4 years, three patients were in remission, and two of these patients had durable complete for more than one year.^271^ Moreover, most of the patients had smaller tumor lesions after receiving TIL treatment. On the first CT scan after receiving treatment, the diameter of the tumor lesions was reduced by an average of 38%.

#### Cervical cancer

In a phase II clinical study of LN-145, a TIL therapy for advanced cervical cancer, most of the enrolled patients had refractory disease after 2–3 prior treatments.^268^ At a median follow-up time of 3.5 months post-infusion, the objective response rate of LN-145 treatment was 44%, and the disease control rate was 85%. Three patients' tumors completely disappeared, and nine patients' tumors shrank significantly. With a median follow-up of 3.5 months, 11 of the 12 patients had sustained responses, and no serious adverse events occurred. Based on the promising data of this clinical trial, the US FDA granted LN-145 with “breakthrough therapy” status for advanced cervical cancer, with approval entering the fast approval track.

#### Metastatic breast cancer

In 2018, a case report was published about a patient with refractory estrogen receptor-positive metastatic breast cancer who received TILs for four mutant proteins (SLC3A2, KIAA0368, CADPS2, and CTSB). Twenty-two months after the infusion, the tumor had completely disappeared, and 4 years later, there was no progression or recurrence.^270^ Larger-scale clinical studies of TIL therapy against breast cancer are currently ongoing.

#### Osteosarcoma

A clinical study was performed to determine the safety and efficacy of TILs and anti-PD-1 therapy for the treatment of osteosarcoma. In this study, 30 patients received anti-PD-1 monotherapy, and another 30 patients received TIL+anti-PD-1 combinational therapy. At the last follow-up assessment, no patients receiving monotherapy survived, with a mean overall survival of 6.6 months. In contrast, 10 of the 30 patients who received combinational therapy survived, with an objective response rate of 33.3%. Of note, two of the 10 patients experienced complete remission according to imaging examination. The mean overall survival was 15.2 months, which was more than doubled that of patients who received PD-1 monotherapy.^272^

#### Ovarian cancer

The combination of ICB with TIL therapy for the treatment of ovarian cancer has been tested in a phase I clinical study. The results showed that one patient achieved a PR, and the other five experienced SD for up to 12 months.^273^ The best strategy for the design of combinational treatment still requires further investigation.

From the clinical applications above, we can infer that the combination of ICB with TIL therapy can be an approach for the future design of immunotherapy. This combinational strategy has been proven for its safety against some types of tumors, with five potential advantages: (1) the killing ability of TILs can be improved by ICB;^274^ (2) TILs are believed to have specific clones that recognize tumor antigens, and advanced technologies have also allowed targeted screening processes to expand antigen-specific TILs and improve specificity;^269^ (3) there is potential for these treatments to be further combined with radiotherapy and chemotherapy to reduce tumor recurrence;^275^ (4) both TILs and ICB antibodies can be reinfused to maintain the antitumor response;^276^ and (5) the other host immune cells can be activated after ICB, which may form synergistic antitumor effects with TILs.^54^ TIL therapy has shown great potential for solid tumors, and new clinical studies have been conducted to expand the scenario of applying TILs to other cancers in the future.^277^

### Engineered TCR T therapy

TCRs are specific receptors on the surface of T cells. By recognizing and binding to the antigens presented by MHC, they can activate the division and differentiation of T cells.^278^ However, not all patients have T cells that can recognize tumors. Therefore, TCR-T therapy involves taking T cells from patients and expanding these cells to equip patients with new TCRs that can recognize specific cancer antigens.

The design of engineered TCRs used for TCR-T therapy is highly dependent on the identification of specific tumor antigens. Some antigens, such as NY-ESO-1, are widely expressed in tumor tissues and can be exploited to develop TCRs to treat different types of tumors.^279^ However, TCRs can be identified and synthesized in a patient-specific manner. As TIL therapy exploits the difference between intratumoural versus systemic lymphocytes, the identification of specific mutations in a patient's tumor guides the generation and application of TCRs that can effectively target these mutations. These TCRs can then be isolated, cloned and expressed on T cells before these engineered T cells are expanded in vitro and reinfused into the patient.^273,280^ This is a highly personalized treatment approach that enhances the specificity of the therapy. TCR therapy has made breakthroughs in the treatment of melanoma and has also achieved certain results in the treatment of liver cancer, breast cancer, and ovarian cancer. However, TCR recognition of tumor antigens requires antigen expression by the MHC molecule, and tumor cells will escape T-cell killing by decreasing the expression of MHC.^56,281^

### CAR-T cell therapy

**Fig. 5:**
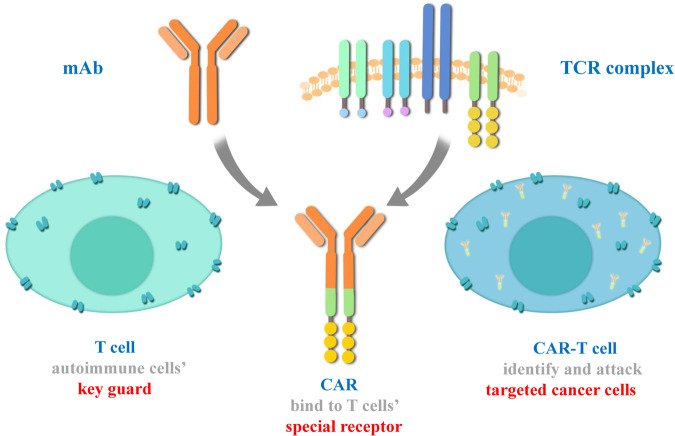
Structure of CAR-T cells

CAR-T cell therapy is another type of ACT strategy.^282^ Sharing a similar principle with TCR T therapy, the patient's T cells are “equipped” with the synthetic CAR, expanded and reinfused into the patient to generate a tumor-specific immune response (Fig. 5).^283–285^ CARs are designed to recognize tumor-associated antigens (TAAs), which are independent of MHC presentation, therefore enabling T cells to recognize cancer cells in an MHC-nonrestricted manner.^286–288^ At present, ACT of CAR-T cells has become one of the main methods of tumor immunotherapy, providing new therapeutic solutions to many types of tumors.^289–292^ Rigorous clinical studies have also allowed researchers to understand the limitations and side effects of this type of treatment, fostering the development of future immunotherapies based on CAR-T cell refinement.

#### Principles and achievements of CAR-T cell therapy

CAR-T cells are generated by expressing tumor-specific CARs on the plasma membrane of T cells. The structure of CARs usually includes three parts: the extracellular antigen-binding domain, the linker/transmembrane domain, and the intracellular signaling domain.^293^ The extracellular antigen-binding region is designed utilizing the sequences of antibodies, ligands and peptides to specifically bind with TAAs. The transmembrane domain is responsible for connecting the extracellular binding domain with the intracellular signal domain and fixing it on the cell membrane.^294,295^ The intracellular signaling region, including the CD3-zeta domain and the costimulatory domain(s), transduces the signal of antigen recognition to the cell, mediating T-cell activation. The evolution of CAR-T cells has undergone four generations. For the first, second and third generation CAR designs, the intracellular signal transduction regions included zero, one and two costimulatory domains, respectively; the fourth generation usually refers to “armored” CAR-T cells with additional expression of immune-stimulatory factors or cytokines to further enhance T-cell activity.^56,282^

**Fig. 6:**
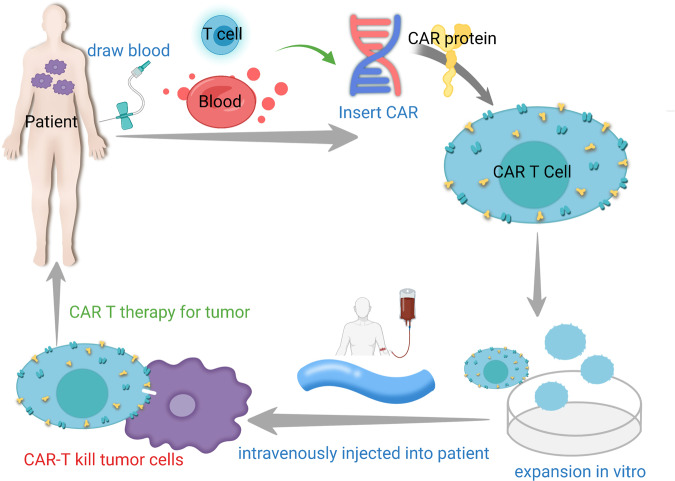
Workflow of CAR-T therapy

CAR-T cell therapy involves the integration of synthetic biology (CAR design), viral technology (CAR transduction) and cell manufacturing (CAR-T cell expansion) (Fig. 6).^296^ The introduction of the CAR generates tumor-specific activation potential in engineered T cells, while ex vivo culture and expansion allow the bypassing of tumor-induced immune suppression.^297–299^ As a result, large numbers of tumor-specific cells are infused back into the patient.^300–302^ CAR-T-cell therapy has shown promising clinical results against several types of cancers. At present, 6 CAR-T-cell therapy drugs have been approved for marketing worldwide, including four targeting CD19 in B-cell leukemia/lymphoma (Kymriah from Novartis; Yescarta and Tecartus from Kite/Gilead; Breyanzi from Bristol-Myers Squibb) and two targeting APRIL or BCMA in multiple myeloma (Abecma from Bristol-Myers Squibb and Cilta-Cel from J&J and Legend Biotech. Of the six products, two have also been approved by the Chinese FDA, including Yescarta (with the name of Achilles by Fosun Kite) and Breyanzi (with the name of Requilense by JW Therapeutic). In addition, there have been almost 1000 clinical trials registered for CAR-T cell treatment against various types of tumors.^303^

#### Challenges of CAR-T cell therapy

Despite the clinical successes listed above, the broader application of CAR-T cell therapy is still complicated by challenges from different aspects. First, when attacking tumor cells, CAR-T cells may cause severe side effects and toxicities that can be lethal. Second, the cytotoxicity of some CAR-T cells is not highly tumor-specific and may cause damage to normal tissue. Third, the manufacturing process of most CAR-T cell products is time consuming, which may result in further deterioration of some patients' tumors during the cell-producing window period. Furthermore, the long-term efficacy of CAR-T therapy against blood cancer still requires long-term follow-up observation, while CAR-T cell therapy application for solid tumors needs further study. These challenges will dictate the development of the entire field of T-cell engineering in the future.

*Cytokine release syndrome (CRS)*, also known as a “cytokine storm”, is the most frequently observed adverse reaction with CAR-T treatment. After CAR-T cell infusions, the systemic inflammatory response can be elicited by the rapid rise of IL-6 and IL-13.^304–308^ The clinical manifestations mainly include fever, fatigue, headache, epilepsy, nausea, chills, and dyspnea. Patients with severe CRS may develop acute respiratory distress syndrome, hypotension, tachycardia, liver damage, renal failure and fulminant hemophagocytic lymphohistiocytosis (HLH), which can all become lethal.^290,309^ CRS usually occurs within a week after CAR-T infusion, with peaks occurring 1 to 2 weeks after infusion. It is worth noting that in the pathophysiological process of CRS, in addition to activated CAR-T cells, endogenous immune cells, such as monocytes, macrophages, and/or dendritic cells, are involved in the synthesis and release of various cytokines and clinical CRS symptoms.^306,307^

*Immune effector cell-associated neurotoxicity syndrome (ICANS)* refers to nervous system toxicity after CAR-T-cell infusions. The incidence of ICANS is closely correlated with CRS, with the rate differing between clinical studies but can be as high as 50%.^310,311^ The symptoms can manifest as mild behavioral abnormalities, unresponsiveness, aphasia, and epilepsy and are more common in patients with B-ALL than in those with other diseases. Mild ICANS is often reversible, but the etiology of ICANS is still unclear and may be related to various factors, such as cytokine release, infiltration of CAR-T cells into the central nervous system, and the dose of CAR-T-cell infusion.^312,313^

*On-target, off-tumor toxicity* is commonly observed in patients with B-cell malignancies after CAR-T therapy, as CAR-T cell targets (CD19, CD20 and/or CD22) are expressed in both normal and malignant B cells. The resulting B-cell aplasia can lead to hypoimmunoglobulinemia, and regular intravenous immunoglobulins can reduce the risk of opportunistic infections.^314,315^ However, the duration of B-cell aplasia is also an indicator of functional CAR-T-cell persistence and superior antitumor response. For CAR-T cells targeting other TAAs, the on-target, off-tumor toxicity needs additional attention. A clinical study using HER-2 CAR-T cells resulted in severe damage to the patients’ cardiac and respiratory systems.^316^ Due to the difficulty in finding TAAs that are exclusively expressed by tumor cells, CAR manipulation may be required to modify the activation potential against different antigen expression densities.^317^

*Uncertain long-term efficacy* is indicated in most clinical studies of CAR-T cells, even against leukemia, where the most promising CAR-T cell clinical response was observed. Several studies have shown that for B-ALL, although the clinical remission rate can reach more than 80%, the recurrence rate within one year can also reach more than 40%. This may be related to the inability of the engineered T cells to persist in the patients due to a variety of immune escape factors expressed by cancer cells, causing T-cell senescence and exhaustion.^318,319^ Furthermore, most clinical studies of CAR-T cell therapy against solid tumors have shown unsatisfactory results, despite some evidence of antitumor activity or even complete responses in some patients.^320^ CAR-T cells targeting solid tumors face challenges including vascular disorders that block T-cell infiltration, limited options for TAAs and tumor heterogeneity, which causes antigen escape. The application of CAR-T cells in solid tumors needs further exploration.^319,321^

#### Strategies of CAR-T cell refinement

##### Leveraging specificity with broad targeting

The long-term antitumor function of CAR-T cells has been complicated by tumor recurrence post-infusion. In view of the risk of antigen escape after CAR-T treatment, the design of a bispecific CAR-T that targets multiple TAAs can be adopted. These CAR-T cells are named “OR-gated”, meaning that the expression of either TAA on tumor cells can elicit CAR-T-cell activation. For B-ALL treated with CD19 CAR-T cells, recurrent malignant cells may downregulate CD19 but maintain CD22 expression. Therefore, “OR-gated” CARs targeting both CD19 and CD22 have the potential to reduce antigen escape.^297^ However, there are huge obstacles to applying “OR-gated” CAR-T-cell therapy in solid tumors.^322,323^ Given that solid tumor TAAs can hardly meet the criteria of stringent tumor specificity, off-target effects can be more significant if CAR-T cells require either of two TAAs for activation.^324–326^ In contrast, designing an “AND-gated” CAR construct, which needs both TAAs to activate T cells, can reduce the probability of on-target, off-tumor effects.^327^ The application of these CAR-T-cell designs will be highly dependent on the nature of the targeted tumors and clinical needs.

##### Enhancement of long-term antitumor effects

While tumor antigen escape accounts for many instances of postinfusion recurrence, quite a few relapsed cases still maintain the expression of the targeted antigen,^328^ indicating that CAR-T-cell dysfunction is an important contributor to treatment failure. Therefore, improving the fitness of CAR-T cells, including the activation potential, proliferation and survival capability, and prolonging the survival time of CAR-T cells in patients is one of the key directions to enhance clinical responses.^329,330^ Intriguingly, most strategies to optimize CAR-T-cell products are focused on preventing the overactivation and subsequent exhaustion and/or apoptosis of CAR-T cells.^331^ The approaches include modification of the manufacturing environment,^332^ pre-enrichment of memory T-cell subsets,^333^ CAR construct engineering to reduce signaling domains,^334^ and combination of small molecules to inhibit activation signals.^335^ Most of these methods have shown responses superior to those of traditional CAR-T therapy in preclinical models, which needs to be further validated for their safety and efficacy in clinical studies.

##### Reduction of manufacturing cost

To date, all approved CAR-T-cell therapies use autologous T cells to generate the therapeutic product. The production of this highly personalized therapy requires high cost.^336^ The CAR-T products approved by Novartis and Kite are priced at 475,000 USD and 373,000 USD, respectively, and the high prices limit their market potential. Novartis' CAR-T products yielded $12 million in revenue in the first quarter of 2018, only 30% of the expected revenue; Kite's CAR-T products also yielded less than expected within two months of approval. In addition to the high cost, the quality and stability of CAR-T-cell therapy have been major concerns. Autologous T cells are inconsistent in their quality and quantity, especially in patients who have been heavily pretreated with radiation and chemotherapy.^337^ The development of allogeneic “universal” CAR-T products aims to address these challenges. The technology has the potential to turn CAR-T cells into “off-the-shelf” drugs, with the advantages of large-scale production, lower cost and consistent characteristics. At present, although most general CAR-T cell therapies are still in the preclinical or early clinical stage, their attractive therapeutic potential is enough to serve as a strong driving force for continued research and development for the future benefit of more patients.^338^

##### Toxicity control

The toxicity and side effects exhibited by CAR-T cell therapy indicate that some control programs need to be developed to regulate the activity of CARs. A large number of methods have been used to control the safety of CAR-T cells; these include the rapid removal of infused cells by installing a suicide switch, which can be controlled by small molecules or antibodies. Commonly used suicide switches include inducible caspase-9 (iCasp9), thymidine kinase (HSV-TK) in herpes simplex virus, and suicide epitopes. However, such a suicide switch clears all therapeutic CAR-T cells, which compromises the antitumor response. Therefore, noncytotoxic reversible systems that do not clear CAR-T cells are under development and have the potential to maintain the balance between maintaining cytotoxicity and controlling toxic responses^307^ (Table 1).

**Table 1:** List of CAR-T therapies available

Product	Company	Approve times	Target	Indications	Price
Kymaiah	Novartis	2017	CD19	B-cell non-Hodgkin's lymphoma that failed first- or second-line therapy Acute lymphoblastic leukemia	$475,000
Tecartus	Gilead	2020	CD19		$373,000
Yescarta	Kite	2021	CD19	Large B-cell lymphoma or follicular lymphoma	$373,000
Breyanzi	BMS	2021	CD19	B-cell precursor acute lymphoblastic leukemia	$410,300
Abecma	BMS and Bluebird Bio	2021	CD19-BCMA	Relapsed and refractory large B-cell lymphoma after second-line or above systemic therapy	$438,000
Akilence	Fosunkite	2021	CD19	Specific non-Hodgkin's lymphoma	¥1,200,000
JCAR014	WuXi Junuo	2017	CD19	Aggressive B-cell non-Hodgkin’s lymphoma (NHL)	–

### NK cell therapy

NK cells are another important type of immune cell that can mediate direct cytotoxicity. Mechanistically, NK cells play a key role in the first line of defense against cancer, mediating antitumor effects through two pathways: direct cytotoxicity through the release of post-perforin and granzyme or death receptors and the regulatory effect by secreting cytokines and chemokines that activate APCs and T cells.^339–341^ Therefore, in addition to drugs targeting the ICs on NK cells, which were discussed earlier in this article, ACT using NK cells is also under rapid development.^342,343^ There are many similarities between ACT strategies built around NK cells and T cells, despite the differences between the innate and acquired immune systems.

Similar to T cells, NK cells can also be transduced to express CARs. The development of CAR-NK cells followed the evolution of CAR-T cell therapy, and CAR-NK cells often directly adopt CAR-T cell designs. In 2020, the first CD19-targeted CAR-NK clinical study with confirmed safety and evidence of efficacy against B-cell malignancies was reported.^344^ Moreover, many preclinical studies have confirmed the antitumor activity of CAR-NK cells targeting other types of tumors.^345,346^

#### Advantages of NK cell therapy

The most important advantage of NK cell therapy relies on the nature of NK cells as part of the innate immune response. Compared with allogeneic T-cell products, allogeneic NK cells are significantly less concerning in terms of GvHD.^347^ Technological advances have also made it possible to expand NK cells in large numbers using feeder cells,^238^ providing a stable resource to manufacture “off-the-shelf” products with more controllable costs.

Because NK cytotoxicity is triggered by “missing-self” recognition, NK cells, in particular, have the capability of killing tumor cells with MHC downregulation. NK cells also have special killing ability to virus-infected cells, making them particularly suitable for the treatment of HPV- or EBV-associated tumors. Recent studies have also found that NK cells can inhibit the formation of tumor-associated blood vessels. In addition, NK cells are the most critical mediator of ADCC and have great potential to be combined with targeted antibody therapy. Furthermore, according to the clinical results of NK therapy, the incidence rates of CRS and ICANS are significantly reduced compared with those of CAR-T therapy,^344^ making it possible for this strategy to be applied in patients with less stringent limitations of age and prior treatment.

#### Challenges of NK cell therapy

Although allogeneic NK cells can provide a sufficient amount of starting material for ACT, freeze‒thaw cycles can significantly reduce NK-cell viability and cytotoxicity. Moreover, the in vivo expansion potential of NK cells is not as robust as that of CAR-T cells, which may lead to tumor recurrence early after infusion. Indeed, in a clinical study of CD19-CAR-NK cells, no correlation was found between the infusion doses and clinical outcome,^344^ indicating the major challenge of sustaining NK-cell activation against tumors.

For CAR-NK cell therapy, most current CARs have been directly adopted from CAR-T cells. The location of the CAR-binding epitope and its distance from the surface of CAR-NK cells may affect cytotoxicity in a T-cell-independent manner. The relatively high number of cells in infusions also makes NK approaches sensitive to insertional mutagenesis caused by viral CAR vectors. The Sleeping Beauty transposon system and mRNA transfection strategy, which have both been successfully applied to CAR-T cell production, remain to be evaluated as practical methods to generate CAR-NK cells.

#### Directions for the future development of NK cell therapy

NK cytotoxicity can be affected by multiple immunosuppressive mechanisms in the TME, including IL-10, indoleamine 2,3-dioxygenase, prostaglandin E2, transforming growth factor beta (TGF-β) and hypoxia.^348,349^ Enhancing NK-cell cytotoxicity and persistence in vivo is believed to be the major direction of advancing NK therapy.

##### Cytokines to support NK-cell maintenance

IL-15 has been identified as a key cytokine that enhances NK-cell activity. In syngeneic mouse models of cancers such as melanoma, colorectal cancer, lymphoma and lung cancer, injection of IL-15 was well tolerated and facilitated the expansion of NK cells. IL-15 can therefore be used as a monotherapy and as an adjuvant for NK-cell adoptive cell therapy. In a study targeting non-Hodgkin's lymphoma, medium and high concentrations of IL-15 effectively improved the survival rate of patients. IL-15 is also the main factor that induces NK-cell expansion in NK-cell culture in vitro.^350^ Therefore, multiple engineered NK-cell designs incorporate the expression of IL-15,^344^ and these designs are currently being tested in clinical studies.

##### Combination with NK checkpoint blockade

In 2011, the anti-KIR monoclonal antibody (lirilumab) was licensed to BMS for late-stage clinical development at a total price of $440 million and then tested in seven clinical trials. CD94/NKG2A-targeting monalizumab from AstraZeneca was soon developed as an NK ICB therapy. Moreover, NK-cell stimulatory receptors, including NKG2D, NCR, CD226, and CD16, provide targets for agonistic antibodies. Of note, most monotherapies targeting NK-cell checkpoints have failed to yield promising clinical responses; therefore, combination with NK-cell ACT might be a strategy for maximizing the stimulatory function of antibodies.

The “off-the-shelf” nature of NK-cell products makes their broad application possible. NK cells can specifically recognize and target cells with MHC downregulation, which can compensate for reduced antitumor T cell function. With more in-depth research on NK-cell activation and maintenance, future therapeutic methods must not only generate tumor-specific NK cells but must also increase their persistence in vivo to enhance their therapeutic potency.

### CRISPR technology advances cellular immunotherapy

**Fig. 7:**
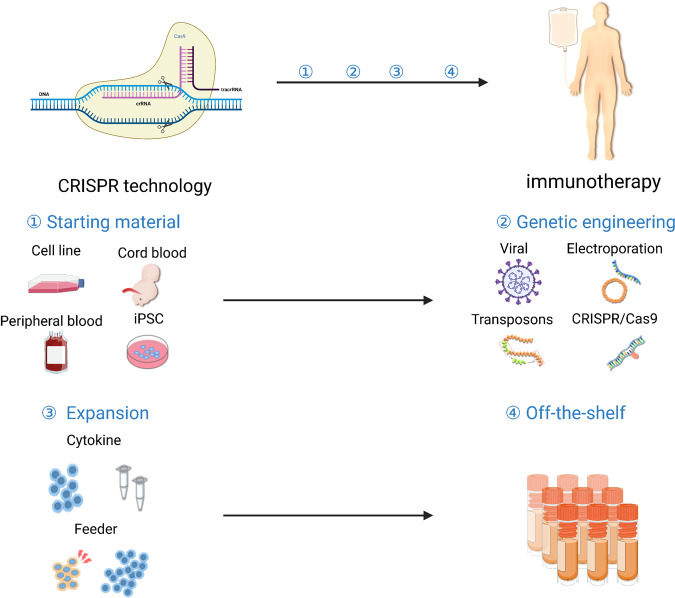
Workflow of CRISPR technology-based immunotherapy

Recently, CRISPR/Cas9 technology has greatly improved our understanding of tumor genomics and contributed to cancer immunotherapy.^351–353^ Using this genome editing system, therapeutic immune cells can be further engineered to enhance tumor recognition and reduce exhaustion (Fig. 7).^350,354–356^ The first clinical study using CRISPR-engineered T cells was initiated in 2016 by Sichuan University in China. In 2020, a clinical study reported the use of PD-1 knockout T cells to treat patients with NSCLC that was refractory to radiotherapy and chemotherapy;^357^ it demonstrated that CRISPR engineering is safe in T cells, which paves the way for combining CRISPR technology with other T-cell modification approaches. NY-ESO-1 TCR-T cell therapy with CRISPR-mediated knockout of TCR and PD-1 represents the first tumor-specific T cells with further genetic modifications tested in the clinic.^358^ Moreover, the CRISPR gene editing system allows for broader application of allogeneic T-cell therapy.^359^ When allogeneic products are depleted of their endogenous TCRs and HLA molecules, they become less likely to be rejected and have a reduction in GvHD potential.^360^ Alternatively, CRISPR-mediated screening systems have been applied in multiple clinical studies. Such screening of tumor cells can identify targets that render tumors more sensitive to T-cell cytotoxicity.^361^ Moreover, screenings performed directly on therapeutic immune cells, such as CAR-T cells, have identified and validated critical factors that can be exploited in future research to further potentiate cellular immunotherapy.^341,362–364^

## Biomarkers for immunotherapy—lessons from ICB and CAR-T cells

### Biomarkers for ICB

The overall impressive clinical effect of ICB has led to several approvals of related treatments. However, not all patients can benefit from ICB treatment, making it critical to identify biomarkers for efficacy prediction. For patients to receive accurate and effective treatment, biomarkers are responsible for screening and classifying patients, accurately identifying patients with drug response, and allowing them to receive the best treatment as soon as possible.^365–367^ Indeed, inappropriate application may even cause disease progression,^368^ illustrating the need for ICB to be prescribed in a personalized manner based on the analyses of certain biomarkers.

PD-L1 was used as the first biomarker for anti-PD-1 treatment, which was included in the prescription guide of pembrolizumab.^369–372^ However, PD-L1 can be induced by interferon and many other immunological signaling pathways during treatment,^373^ which undermines the utilization of PD-L1 as a predictive biomarker for ICB. The first reported study of acquired resistance to anti-PD-1 ICB identified mutations involved in interferon and antigen presentation pathways, which have become critical biomarkers to predict relapse post ICB.^374^ Further studies have identified additional mutations and immunosuppressive molecules that are associated with a poor prognosis in ICB-treated patients.^375–377^ In contrast, the T-cell inflammatory gene expression profile (GEP) and somatic copy number variation (SCNA) are correlated with a good prognosis in ICB-treated patients.^378,379^

At present, common or potential biomarkers related to immunotherapy efficacy have been reported mainly in the following categories based on their accessibility: (i) surface markers, including PD-L1 and some other inhibitory receptors, which can be examined by immunohistochemistry of tumor tissues; (ii) genetic biomarkers, such as tumor mutation load (TMB), mismatch repair system deficiency (dMMR), high microsatellite instability (MSI-H), neoantigens and mutations of the antigen presentation pathway, which all require genomic analyses of the tumor; and (iii) circulating tumor DNA (ctDNA), which is accessible by analyzing peripheral blood.^380–382^ Some of these biomarkers have been verified by phase III clinical trials and are widely used in the clinic.^383–385^ More biomarkers reflecting immune efficacy are still under continuous research and testing.^386^ One good example was the study from Sun Yat-sen University Cancer Hospital, which was a comprehensive analysis of genomic data identifying POLE and POLD1 gene mutations that can be used as independent biomarkers for predicting the efficacy of immunotherapy across cancers, which can provide more accurate guidance for the clinical application of immunotherapy^384^ (Table 2).

**Table 2:** List of biomarkers for immunotherapy

Biomarker	Origin	T cell source (Y/N)	Target of immunotherapy (Y/N)	Clinical acceptance
PD-L1	Tumor tissue	N	Y	Broad
TMB	Tumor tissue	N	N	Broad
MSI-H	Tumor tissue	N	N	Limited
dMMR	Tumor tissue	N	N	Limited
cDNA	Plasma	N	N	Limited

#### MSI-H and MMR

MSI-H refers to the variation in the length of short and repeated DNA sequences, which may include insertions, deletions or mutations caused by MMR functional defects.^387–389^ The MSI phenomenon was first found in colorectal cancer in 1993. According to the degree, it can be divided into the following: MSI-H, MSI-L and microsatellite stability.^389,390^ Mismatch repair (MMR) is a DNA damage repair mechanism against the wrong insertion, deletion and mismatch of bases that may occur in the process of DNA replication or recombination.^391,392^ The system consists of a series of specific DNA mismatch repair enzymes, which usually depend on four key genes: MLH1, PMS2, MSH2 and MSH6. Germline depletion of MMR genes is the “gold standard” for the diagnosis of Lynch syndrome.^393^ Due to the functional inactivation of MMR genes, patients with Lynch syndrome often simultaneously show MSI-H status and MMR defects (dMMR), which are also shared by some tumors.^394^

At present, it is recognized that dMMR/MSI-H is used as a prognostic factor for stage II colorectal cancer. For stage II colorectal cancer patients with the dMMR/MSI-H phenotype, grade 3/4 differentiation (low differentiation) is not considered a high-risk factor.^393,395^ Regarding ICB treatment, multiple clinical studies have shown that PD-1 antibodies can lead to survival benefits in patients with dMMR/MSI-H tumors.^396,397^ In May 2017, pembrolizumab was approved for solid tumor patients with MSI-H or dMMR who have progressed after previous treatment and have no satisfactory alternative treatment.^386,396,398^ In 2017 and 2018, the FDA successively approved the treatment of metastatic colorectal cancer patients with MSI-H or dMMR after treatment with fluorouracil, oxaliplatin and nivolumab alone or in combination with ipilimumab. Therefore, MSI-H and dMMR can be used as primary screening methods.^399^

#### TMB

TMB refers to the total number of mutations, base substitutions, and insertion or deletion errors detected per million bases.^400,401^ A number of clinical studies have confirmed that patients with high TMB tumors are more likely to benefit from ICB treatment.^402–404^ This correlation was found in tumors that generally have high immunogenicity, such as melanoma, urothelial cancer and NSCLC, as well as colorectal cancer, which has different degrees of immunogenicity between individuals.^405^ In the analyses of clinical and preclinical studies, TMB was also found to be associated with tumor T-cell infiltration and an “inflamed” TME and may be related to the high expression of immunoreactive neoantigens in these tumors.^406–408^

#### Neoantigens

Neoantigens are proteins that are specifically expressed only in tumor cells and can be recognized and killed by T cells of the immune system. During the development of tumor cells, nonsynonymous mutations change the amino acid coding sequence, causing tumor cells to express abnormal proteins in a tumor-specific manner. These proteins may also activate the immune system and lead to an attack by the immune system on tumor cells. These antigens from abnormal proteins that can be recognized by immune cells are neoantigens. Neoantigens have two major characteristics: first, they are unique to tumor cells and are not found in normal tissues or cells; second, these antigens should have corresponding TCRs that recognize them specifically.^409^

Neoantigens can be ideal biomarkers for ICB if clearly defined as “immunogenic neoepitopes”, which will reflect the extent of tumor immunogenicity with more accuracy than MSI status, MMR status and TMB level, so if neoantigens that bind with high affinity to MHC can be produced, the possibility of an immune response will be higher. However, it remains challenging to validate the “quality” of neoantigens, which refers to their capability to elicit immune recognition and activation.^410^ Currently, neoantigens are mostly used to support other biomarkers. For example, melanomas with TMB>10 often produce neoantigens with high frequency and are sensitive to PD-1 inhibitors; in contrast, melanomas with TMB<1 are unlikely to generate neoantigens and are insensitive to PD-1 inhibitors. The direct utilization of neoantigens as biomarkers would rely on the development of assays and algorithms that can precisely detect both the quantity and quality of neoantigens within a tumor.^411^

#### Circulating tumor DNA (ctDNA)

The DNA in the cell can sometimes dissociate into the blood, forming circulating DNA. ctDNAs are fragments derived from four sources: necrotic tumor cells, apoptotic tumor cells, circulating tumor cells, and exosomes secreted by tumor cells.^402^ By detecting gene mutations in blood ctDNA, we can understand the changes in tumor cells in the body in real time, thus providing a clinical basis for tumor treatment and prognosis.^403,404^ A research team from the Princess Margaret Cancer Center in Canada conducted a prospective phase II clinical trial in patients with 5 different types of advanced solid tumors who were treated with pembrolizumab. Analysis of the correlation between changes in ctDNA levels and immune efficacy after treatment revealed that ctDNA levels were associated with the clinical response to ICB.^412^ Another research group also proved that ctDNA could be a good biomarker for the immunotherapy response in different types of cancers. Therefore, ctDNA has the potential to become an easily accessible biomarker used for screening and prediction in a cost-effective manner.^413,414^

### Biomarkers for CAR-T-cell therapy

Although the initial CR rate of CAR-T-cell therapy against B-cell leukemia can be as high as 90%, a significant proportion of patients develop tumor recurrence.^328^ Furthermore, not all patients with lymphoma or multiple myeloma achieved satisfactory response with CAR-T cell therapy, warranting the discovery of biomarkers that can help classify specific cohorts of patients who can benefit from this type of treatment. To date, no biomarkers have been utilized to guide the enrollment of patients, but some CAR-T cell intrinsic and extrinsic factors have shown intriguing correlations with the therapeutic response.^415,416^

#### Tumor antigen expression

The nature of CAR-T cells as a targeted therapy requires the expression of TAA on tumor cells to elicit T-cell activity, which is indeed the most critical biomarker for CAR-T cell efficacy. As tumor antigen escape is a major mechanism of tumor recurrence post CAR-T cell therapy, some studies have also revealed that downregulation, instead of complete loss of TAA, inhibits CAR-T cell function.^417^ While CAR-T cells can be designed to increase their sensitivity to low-level TAAs, the TAA expression density might become a predictive biomarker.^417^

#### Product characteristics

The inconsistent quality of therapeutic products has been a major challenge of autologous CAR-T cell therapy. The starting materials from patients can be significantly altered by the many lines of prior treatment. As a result, the composition of T-cell subsets showed more variation across patients with tumors compared with healthy donors. Therefore, the difference in product quality is an important contributor to clinical outcomes. In 2018, Kite Pharma evaluated the polyfunctionality of their CAR-T-cell products, which reflects the capability to produce multiple cytokines at the single-cell level and correlates with the therapeutic effect against lymphoma. Some other studies profiled the phenotypes and transcriptomes of the infusion products, identifying a memory-like population enriched in the products that ultimately lead to superior responses. With ongoing validations in larger-scale studies, these product characteristics can be exploited as useful biomarkers to predict the clinical response before infusion.^317^

## Other types of immunotherapies

### Tumor vaccines

Preventive tumor vaccines can prevent the development of certain cancers, including the HPV vaccine against cervical cancer, vaginal cancer, vulvar cancer, anal cancer and condyloma acuminatum and the HBV vaccine to prevent liver cancer.^418–420^ Therapeutic tumor vaccines involve the injection of tumor antigens in the form of free peptides or peptides loaded on APCs to activate immune cells to restore their autonomous antitumor ability. In preclinical models, therapeutic tumor vaccines have been confirmed to prevent cancer growth and metastasis and reduce relapse after the termination of other types of treatment.^414,421,422^ Tumor vaccines are mainly divided into the following four types: tumor whole-cell vaccines, genetically engineered vaccines, protein peptide vaccines, and dendritic cell vaccines.^423–425^

Dendritic cells (DCs) were first discovered by the Canadian scientist and 2011 Nobel Laureate Dr. Ralph M. Steinman. Dendritic cells are a heterogeneous group of innate immune cells with antigen-presenting functions and are considered the only immune cell type that can activate naive T cells.^426^ In the context of cancer, however, the number and vitality of DCs are not enough to trigger sufficient T-cell activation against malignant cells. Therefore, DCs can be isolated from cancer patients, primed and loaded with tumor antigens in vitro. The resulting dendritic cells express tumor antigens on their surface, and the activated DCs are then used to initiate an immune response, which is referred to as a “DC vaccine”.^427,428^ Unfortunately, DC isolation, priming, and antigen loading can be complex, time consuming, and labor intensive, which limits the capacity of DC vaccine application. However, the immune response elicited by tumor antigen-primed DCs is highly tumor-specific with limited side effects.^416,429^ Dr. Ralph M. Steinman also benefited from the DC vaccine, which extended his lifespan from an expected few months to four and a half years with refractory pancreatic cancer. On 29 April 2010, the US FDA approved a therapeutic tumor vaccine, sipuleucel-T from Dendreon, for treating advanced prostate cancer.^430,431^ With the continuous progress of science and technology, a variety of tumor vaccines have gradually entered the clinic.

### Neoantigens and immunotherapy

Neoantigens are protein fragments present on cancer cells, offering a novel way to achieve cancer cell-specific targeting.^432^ Neoantigen vaccines are individualized based on a patient's specific tumor profile. Interest in the field has grown since the first human clinical trials using the neoantigen vaccine began in 2015.^433,434^ Despite subtle differences between platforms, the general steps of making neoantigen vaccines are mostly conserved; they include (1) tumor biopsy, in which tumor samples are taken from patients for genomic purification; (2) whole-exome sequencing of tumor cells and normal cells, which allows researchers to search for unique mutations in tumor cells; (3) prediction and selection of specific neoantigens as targets; and (4) development of personalized vaccines, which is based on predicted neoantigens and can be achieved using various approaches, including peptides, mRNA and DCs.^435–440^ The most critical and challenging step has been the identification of patient-specific neoantigens. While some platforms are focused on developing predictive algorithms to achieve greater accuracy, others use in silico prediction together with functional tests to ensure that the neoantigen indeed triggers immune cell activation.^441^ While the latter enables the validation of targets, it can be extremely time-consuming and expensive. Technical advances are therefore warranted before the application of neoantigen vaccines to larger-scale clinical studies.^431^

### Oncolytic virus

Cancer patients with additional virus infection often experience worsening disease.^442,443^ However, viruses can also be modified to specifically target cancer cells. These “oncolytic viruses” are generated by genome editing and large-scale screenings, the readout of which includes the lysis ability against cancer cells while sparing normal cells. The resulting oncolytic virus candidates can replicate and subsequently lyse tumor cells, which releases more viral particles into the tumor sites.^444,445^ Therefore, a small dose of virus can be expanded in vivo. Talimogene laherparepvec (OncoVex, T-VEC) is an oncolytic virus agent approved by the FDA for use in melanoma in 2015.^446,447^ T-VEC is a type I herpes simplex virus and is an oncolytic immunotherapy preparation based on herpes simplex virus. Herpes simplex virus is genetically edited to help the virus evade the immune system, allowing the modified virus to replicate in cancer cells in a targeted manner. On the one hand, it can directly lyse cancer cells, and on the other hand, it can activate the human immune system by releasing antigens inside the tumor and priming “bystander” immune cells.^448^

Oncolytic virus therapy has demonstrated great potential in combination with other types of cancer immunotherapy. With their capability to mediate tumor antigen spread, oncolytic viruses can lead to an increase in lymphocytes infiltrating the tumors, which enhances the antitumor efficacy of ICB treatment. Another approach is to use oncolytic viruses as delivery vehicles in combination with cellular immunotherapy. With additional genetic engineering, the cytolytic function of the viruses can be suppressed while allowing for the expression of synthetic molecules (i.e., truncated CD19 as a CAR-T cell target). Combining these viruses with CD19-targeted CAR-T cell therapy can thus achieve homogenous expression of TAA and overcome the challenge of tumor antigen escape.^449^

## Targeting the suppressive TME

### Macrophages

These “soldiers” of the innate immune system remove damaged, senescent, and dangerous cells, but in cancer, macrophages facilitate their immune escape and have become an important field of drug development. While early researchers developed treatments by modulating the interactions between tumor cells and macrophages, this complex field of biology has been rewritten, and the application of macrophage therapy has slowed. The great progress of genetic engineering provides a greater possibility to use synthetic biology to redirect macrophages to fight tumors.^450,451^ Several researchers from Carisma Therapeutics in the United States and the University of Pennsylvania published a review titled “Macrophage-based approaches for cancer immunotherapy” in cancer research, outlining the progress made in macrophage immunotherapy and the impact of chimeric antigens. The rise of somatic macrophage therapy.^452,453^

#### Macrophages in cancer

**Fig. 8:**
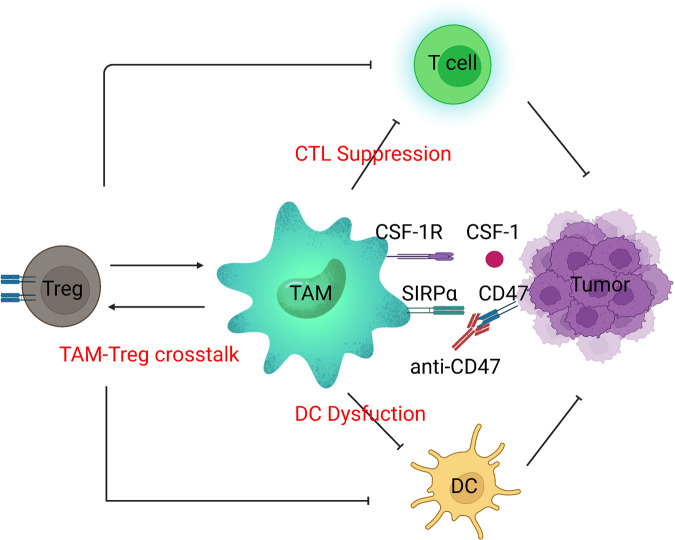
Characteristics of M1 and M2 macrophages (Nature Reviews Immunology)

Macrophages have a variety of functions, including removing cellular debris and pathogens and regulating inflammatory responses. Macrophages are also highly plastic cells that can switch from one phenotype to another depending on microenvironmental stimuli and signals.^454^ The activation state of macrophages is usually divided into two categories: M1-type macrophages and M2-type macrophages (Fig. 8).

Certain M2 macrophage subsets are involved in promoting tumor progression and mediating immune suppression.^455^ Mechanistically, it has been found that tumors recruit monocytes and macrophages to the TME and polarize them to the M2-like phenotype. The central goal of macrophage-targeted cancer therapy is to reprogram tumor-associated macrophages (TAMs) into the proinflammatory (antitumor) subtype, which can be achieved in two ways: reducing the number of M2-like TAMs and/or restoring the antitumor function of TAMs within the TME.^456^ Advances in technology, such as single-cell sequencing, have allowed researchers to see different macrophage subsets with multiple complex biological functions in different TMEs and gain a deeper understanding of the relationship between macrophages and tumor immunotherapy.^457^

#### Inhibitory and stimulatory molecules on TAMs

The most established approach to target TAMs is the blockade of the colony-stimulating factor-1 (CSF-1, also known as the M-CSF)/CSF1R axis. This approach reduces the number of TAMs, which can also be associated with the repolarization of TAMs toward the M1 phenotype.^458^ However, the leading CSF1R inhibitor, cabiralizumab from Five Prime Therapeutics, did not show promising clinical responses in a series of clinical trials,^459^ indicating that the potency of CSF1R inhibition needs to be revisited.

The tumorigenic function of TAMs can also be mediated by TGF-β, an anti-inflammatory molecule normally expressed by macrophages during injury repair. Blockade of TGF-β and concurrent treatment with a STING agonist in a mouse model resulted in tumor regression by upregulating the expression of type I interferons.^455,460^

Toll-like receptors (TLRs) are involved in innate immune sensing. TLR agonists can increase monocyte recruitment/infiltration and induce macrophage repolarization toward the proinflammatory phenotype.^461^ TAMs also express CD40, and CD40 agonists can prevent tumor growth and attenuate drug resistance.^462,463^

These inhibitory (CSF1R, TGF-β) and stimulatory (TLRs, CD40) molecules can all be exploited as targets to restore the proinflammatory function of TAMs. However, the TME is composed of numerous immunosuppressive cells with functional redundancy, which may result in the clinical observation that targeting a single cell type will not lead to sufficient TME alterations to eradicate tumors.

#### CD47

CD47, also known as integrin-related protein, belongs to the immunoglobulin superfamily, which regulates cell proliferation, migration and apoptosis by binding to signal regulatory protein α (SIRPα) on the surface of macrophages or dendritic cells.^464^ CD47 is overexpressed on the surface of most tumor cells as a “do not eat me” signal that escapes phagocytosis by macrophages. Blocking the CD47/SIRPα pathway can induce the phagocytotic function of macrophages to target tumors, which was demonstrated in mouse xenograft models.^465^

Forty Seven was the company that developed the first-in-class CD47 antibodies. After its acquisition by Gilead Sciences, a phase Ib clinical study of the CD47 antibody magrolimab (Hu5F9-G4) was launched. The results showed that magrolimab combined with azacitidine in patients with myelodysplastic syndrome (MDS) reached an overall response rate of 92%.^466^ In addition, another clinical study showed that magrolimab combined with rituximab resulted in an overall response rate of 90% in lymphoma patients.^467^ These results have led to many additional drug candidates targeting the CD47/SIRPα pathway, such as TTI-621/622, ALX148, and OSE-172 (Table 3).

**Table 3:** Variety of drugs targeting macrophage therapy

Company	Candidate	Target	Status
Gilead (forty seven)	Magrolimab	CD47	Phase II
Trillium	TTI-661; TTI-662	CD47	Phase I
ALX oncology	ALX148	CD47	Phase I
I-Mab	TJC-4	CD47	Phase I
Innovent biologics	IBI188	CD47	Phase I
Arch oncology	AO-176	CD47	Phase I
TG therapeutics/novlmmune	TG-1801	CD47	Phase I
BMS/celgene	CC-95251	SIRPα	Phase I
OSE immunotherapeutic	OSE-172(BI-765063)	SIRPα	Phase I
Alector	AL008	SIRPα	Preclinical
Gilead (forty seven)	FSI-189	SIRPα	Preclinical

TTI-621/622 are two CD47 inhibitors developed by Trillium Co.; they are SIRPa-Fc fusion proteins coupled with IgG1 and IgG4, respectively. IgG4 showed slightly weaker binding to Fc receptors on immune cells, which may compromise the potency but enhance protection against CD47-expressing nontumor cells.^468^ was developed by ALX Oncology and contains two SIRPα high-affinity CD47 binding domains linked to the inactive Fc domain of human immunoglobulins. Its Fc domain has been reengineered to inhibit Fcγ receptors. ALX148 is in phase I studies in patients with solid tumors and lymphomas in combination with a variety of chemotherapy agents.^469^ OSE-172 is a monoclonal antibody developed by OSE Immunotherapeutics that targets SIRP-α. In addition, it was designed not to bind with SIRP-γ, which plays a role in the migration of T cells in tissues. Therefore, this antibody will not inhibit the infiltration of T cells. OSE-172 is currently being tested in a phase I clinical study.^470^ In addition to single-drug applications, bifunctional antibodies with CD47/PD-L1, CD47/VEGF and other targets are under development, and we look forward to more studies to bring us more surprising drugs.^471,472^

Despite the clinical efficacy, the toxicity of CD47-targeted treatment cannot be ignored. CD47 is ubiquitously expressed on blood cells, which significantly compromises tumor specificity.^463^ The clinical study of magrolimab found that the number of red blood cells, hematocrit and hemoglobin decreased on the 2nd day of application of the drug and dropped to the lowest point on the 5th to 7th day. Although it was claimed that such a level of anemia can be relieved by supportive care and blood counts can return to normal within 2–3 weeks,^473^ prevention of anemia has been the key focus of toxicity management after anti-CD47 therapy. Magrolimab uses IgG4 for its Fc fragment to avoid excessive killing of red blood cells and has been applied in a low-dose induction manner during clinical application. When a low dose of antibody was given, mild anemia was induced, which subsequently accelerated hematopoiesis in the bone marrow, with the hope of inducing resistance to higher doses of medication.^474^ The clinical safety of this approach remains to be investigated in larger cohorts of patients.

Additional “do not eat me” signals may contribute to the acquired resistance of anti-CD47 therapy, and new targets for macrophages continue to emerge. CD24 is another “do not eat me” signaling molecule that is overexpressed in several types of human cancers, and its receptor Siglec-10 is expressed on TAMs.^475^ CD24 and other molecules with similar functions are emerging as new targets for reprogramming TAMs for immunotherapy.

### Engineering macrophages

Macrophages have a long history of being exploited in ACT. In the late 1980s, Andreesen's group in Germany enrolled 15 patients. Monocytes were harvested from the patients, cultured in autologous serum for seven days, and “educated” with IFN-γ to differentiate into M1-like macrophages.^476^ Of the seven patients with peritoneal carcinoma, ascites disappeared in two of the seven patients who received intraperitoneal macrophage injection. Crucially, other than low-grade fever and abdominal discomfort after intraperitoneal injection, no other side effects were reported. These nonengineered, IFN-γ-activated macrophages have been considered a clinically safe agent but have limited efficacy. One of the main underlying reasons is that in the absence of tumor specificity, an IFN-γ-stimulated M1 phenotype can easily transform into the M2 phenotype in the TME. These challenges suggest that the development of macrophage therapy requires the addition of targeted activating receptors and a more durable approach to M1 macrophage polarization.^476^

**Fig. 9:**
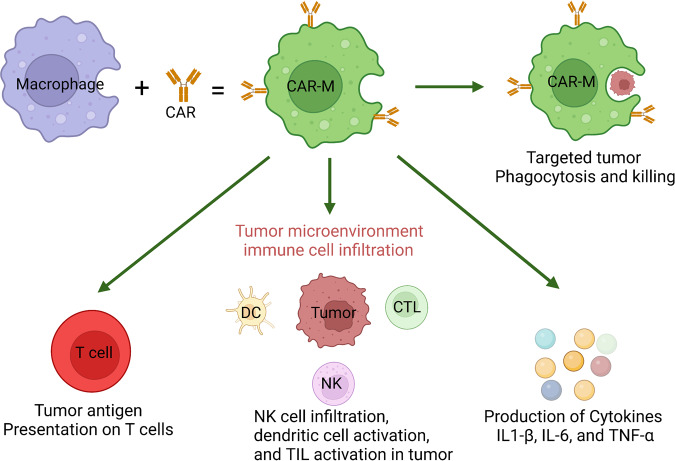
Multipotent antitumor mechanisms of CAR-M therapy

To solve these problems, the use of genetic modification to enhance the antitumor ability of macrophages has gradually attracted more attention. One straightforward strategy is the depletion of inhibitory signals such as SIRPα. The antitumor efficacy of SIRPα-depleted macrophages has been preliminarily shown in combination with radiotherapy.^477^ Another approach is to engineer macrophages to express CARs (CAR-Ms) (Fig. 9). The first challenge of CAR-M generation is the difficulty of transducing macrophages. In 2016, it was reported that the chimeric adenoviral vector efficiently transduced macrophages.^478^ The resulting CAR-Ms, generated with the Ad5f35 vector, eliminated tumor cells more efficiently than macrophages with M1-oriented differentiation. CAR-Ms also induced proinflammatory features of the surrounding TME. The presence of M2 macrophages did not affect the tumor-killing ability of CAR-M cells, highlighting their resistance to TME immunosuppression. In addition, CAR-Ms exhibited a stronger T-cell-stimulating ability and were able to present antigens to T cells after phagocytosis, recruiting resting and activated T cells to tumors.^479^

As part of the innate immune response, macrophages can also be applied in an allogeneic manner; therefore, CAR-Ms have advantages as readily available products. Recent studies have also generated CAR-Ms from induced pluripotent stem cells, which can be further exploited to generate CAR-M banks as “off-the-shelf” products.^480^ CAR-Ms can also be combined with other macrophage-targeted therapies, such as anti-CD47.^481,482^

### Targeting myeloid-derived suppressor cells (MDSCs)

MDSCs originate from hematopoietic stem cells (HSCs) as a result of altered myelopoiesis.^483^ This transient myelopoiesis is terminated after the stimulus is removed, and myeloid cell homeostasis is then restored.^484^ However, in chronic inflammation, cancer, and autoimmune diseases, persistent myelopoiesis may occur to prevent widespread tissue damage of the host, constantly generating IMCs. These cells have distinct characteristics, such as immature phenotype and morphology, relatively weak phagocytic function, and anti-inflammatory and immunosuppressive functions,^485^ matching their descriptive name.

In the 1970s, it was found that abnormal myeloid cells had inhibitory effects on other immune cells. After that, the surface markers Gr-1 and CD11b were adopted to define these immunosuppressive myeloid cells, especially in tumor-bearing mice.^486^ In humans, these myeloid cells are phenotypically characterized by the expression of CD34, CD14 and CD15 and functionally characterized by their ability to suppress T-cell activation.^487^ The term MDSCs indeed refers to a group of heterogeneous cells, which can be roughly divided into granulocytic (G-MDSCs or PMN-MDSCs) and monocytic (M-MDSCs) subtypes. Recent studies using single-cell profiling have uncovered the complexity of MDSC populations, which may differ between disease conditions and even individuals.^485^ Accumulating evidence indicates that the presence of MDSCs is one of the basic characteristics of tumor progression.^488^

**Fig. 10:**
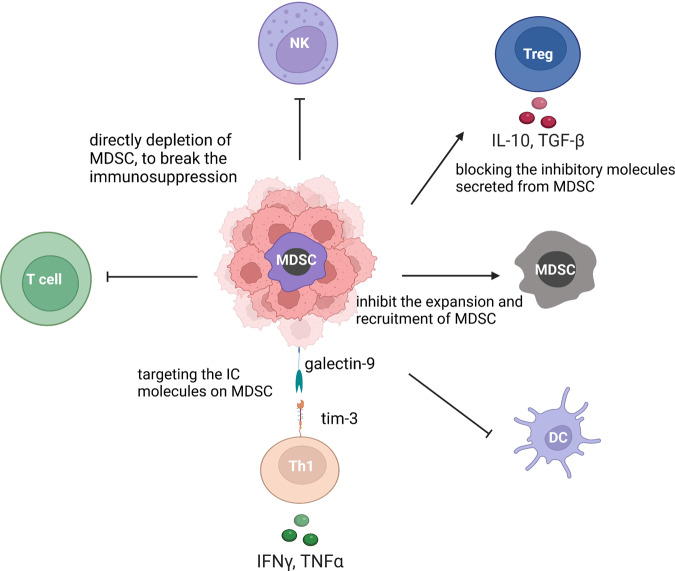
Function of MDSCs

MDSCs have been believed to be one of the most critical obstacles against effective cancer immunotherapy, which has also inspired research into therapeutic strategies targeting these cells. MDSCs exert multiple functions to influence T cells, Treg cells, DC cells and NK cells (Fig. 10). In the early 2000s, before the term MDSC was generated, vitamin D and all-trans retinoic acid (ATRA) were shown to reduce immature myeloid cells in patients with head and neck squamous cell carcinoma and metastatic renal cell carcinoma, respectively.^489^ Currently, MDSC-targeted therapy can be broadly classified into five types: (i) therapies that inhibit the expansion and recruitment of MDSCs; (ii) therapies that restore normal myeloid differentiation; (iii) therapies that target the IC molecules on MDSCs; (iv) therapies that block the inhibitory molecules secreted from MDSCs; and (v) therapies that directly deplete MDSCs.^480,490^ Since the enrichment and activation of MDSCs appear to be universal features of different malignancies, targeting these cells may have broader application potential. It is also intriguing to combine MDSC-targeted therapy with existing chemotherapy or immunotherapy agents, which have early evidence of clinical responses.^490,481^

### Targeting B cells

#### Tumor-associated B cells (TABs)

B cells, which are responsible for antibody production, have long been overlooked for their functions in tumor immunity. However, a recent study showed that similar to macrophages, B cells in the context of tumors, named TABs, display both pro- and anti-inflammatory functions, and the abundance of anti-inflammatory B cells is correlated with the resistance of melanoma to ICB treatment.^491^ However, there are not yet strategies to specifically deplete anti-inflammatory TABs. As proinflammatory TABs can attract effector T cells to tumors, depletion of all TABs has been believed to reduce, rather than induce, the antitumor immune response.^492^ TABs are enriched in tertiary lymphoid-tissue structures (TLSs) within tumor tissues, where they can be activated to recognize cancer cells. NSCLC patients with high levels of B cells within TLSs in their tumors were more likely to respond well to immunotherapy.^493^ It is still only partially understood how the numbers and characteristics of TABs differ across different tumor types. How B cells can be reprogrammed by tumors to inhibit T-cell activation is another topic that requires in-depth investigation.

#### Engineering B cells

As they have the capacity of antibody production, B cells can be upgraded into “biofactories”. Immusoft developed the Immune System Programming (ISP) platform, which can engineer B cells and differentiate them into plasma cells for ACT. The cells will efficiently secrete tens of thousands of personalized antibodies, enabling sustained delivery of therapeutic proteins.^494,495^ Walking Fish Therapeutics is another company focused on B cells, aiming to develop B-cell therapies for cancer, rare diseases, and autoimmune diseases, as well as regenerative therapies and recombinant antibodies.

### Reversing the inhibitory signals from the TME

#### IDO

IDO is a tryptophan-metabolizing enzyme that can convert tryptophan to kynurenine and is overexpressed in multiple types of tumors.^496^ The tumor suppressor gene BIN1 negatively regulates the expression of IDO. In mouse models, the depletion of BIN1 has been confirmed to induce IDO expression and immune suppression in tumors.^497^ IDO can enhance the motility of cancer cells and inhibit the proliferation and function of tumor-targeting T cells.^498^ Mouse xenograft models show that treatment with IDO inhibitors can significantly increase the level of T cells and have a significant tumor-suppressive effect. IDO-targeted drugs have shown efficacy as monotherapies in preclinical models but can be more effective in combination with ICBs targeting CTLA-4 or PD-1/PD-L1.^499–503^

There are mainly four small molecule inhibitors targeting IDO that are currently undergoing clinical research: indoximod, navoximod, epacadostat and BMS-986205. In the reported phase I study of epacadostat, 52 patients with advanced solid tumors were enrolled. Unfortunately, no objective response was observed, but seven patients had stable disease for more than 16 weeks.^502^ In another phase I/II clinical study, epacadostat was combined with pembrolizumab, and a total of 54 patients with solid tumors were included. The overall response rate was 57%.^493^ However, an ECHO-301 clinical study suggested that epacadostat combined with pembrolizumab did not improve PFS in patients with advanced melanoma.^504^ Currently, a number of phase III clinical studies of epacadostat were closed without a satisfactory outcome, which also resulted in the termination of phase III clinical studies of BMS-986205 and indoximod. The enrolled patients were not stratified based on IDO1 expression levels, which may lead to the underestimation of efficacy. However, these terminated clinical studies indicate a lack of understanding of the underlying mechanism between IDO and various immune cells.^504^ IDO inhibitors have been combined with anti-CTLA-4, chemotherapy and other ICs. More types of drugs and larger clinical studies of inhibitors are underway, and the collection of more data will reveal the true antitumor effect of IDO-targeted drugs.^505^

#### IL-41

To explain the limited clinical efficacy of IDO inhibitors, some researchers hypothesized that there may be other activation pathways for aryl hydrocarbon receptors. From an analysis of 32 different types of tumors from the TCGA database, it was discovered that tumors with high expression of IDO1 or IDO2 also had high levels of aryl hydrocarbon receptors.^506^ A more in-depth study identified IL-41 with the highest occurrence rate among the aryl hydrocarbon receptor-related modules. IL-41 was later proven to enhance tumor aggressiveness and suppress antitumor immunity. Targeting IL-41 may be a new avenue of immunotherapy, as demonstrated in preclinical models,^507^ which needs to be validated in clinical studies.

#### Adenosine

Adenosine is an essential component of RNA synthesis. However, adenosine has also been shown to inhibit T-cell functions in the TME.^505^ CD39 is an enzyme involved in extracellular adenosine production and is highly expressed in various human tumors.^506–508^ Some tumor cells exhibited CD39 overexpression compared to normal cells. Moreover, multiple cell types with elevated CD39 expression levels in the TME are vascular endothelial cells, fibroblasts, and some immune cells.^494,495,509,510^ CD39 has been found to play an important role in a variety of immune cells. In macrophages, CD39 acts as a “molecular rheostat” that controls inflammation and regulates the balance between macrophage differentiation.^511–513^ In Treg cells, CD39 enhanced immunosuppressive activity. CD39 expression in MDSCs is positively correlated with NSCLC tumor stage, but its function remains unknown.^511^ Due to its important role in the TME, CD39 has become an emerging target for researchers to develop tumor immunotherapy.^512,513^ In vivo and in vitro data suggest that targeting CD39 sensitizes tumors to PD-1/PD-L1 ICB treatment. Therefore, CD39 therapy combination with ICB might become a new type of tumor immunotherapy.^506,514^

## Conclusion and perspectives

Immunotherapy has become the “fourth bullet” of antitumor treatment following surgery, chemotherapy and radiotherapy. Here, we introduced the history as well as the different classifications of immunotherapy. Early studies to dissect the immune system have allowed the utilization of specific cells to achieve antitumor immunity. The clinical success of ICB and CAR-T cell therapy has revealed the potential that the “edited” immune system in patients with tumors can be “reprogrammed” to fight against malignant cells, which has been further highlighted by many ongoing preclinical and clinical studies targeting almost every type of immune cell.

Despite great success in the clinic, tumor immunotherapy is facing challenges including toxicity control and a low response rate. Although adverse events associated with immunotherapy may indicate the activation of the immune system, severe toxicity is sometimes lethal. Therefore, toxicity control of immunotherapy is not expected to compromise the therapeutic benefit, which is reminiscent of the efforts in controlling GvHD in allogeneic bone marrow transplantation while maintaining the graft-versus-leukemia effect. Moreover, immunotherapy is able to mediate long-term disease-free survival in some patients, but extending the therapeutic potency to more individuals is another barrier against its broader application.

With all emerging novel checkpoint inhibitors and different novel technologies, there is always one critical question for cancer immunotherapy: do patients respond or not respond? It is important to have good biomarkers to predict the response, and it is also important to seek a clinical strategy to improve the clinical response rate.

With a deeper understanding of the mechanisms underlying every type of therapeutic agent, there is also the potential of combinational treatment with chemotherapy, radiation or other types of immunotherapies. PD-1 (PD-L1). Only a small proportion of cancer patients have high TMB and other clinical biomarkers; therefore, the population for clinical application is very limited. Moreover, the therapeutic effect is sometimes not as good as expected. It has been confirmed that the response rate of immune drug monotherapy is low, only 10–30% in most solid tumors. Based on the response limitations, immune combination therapy has become a new research hot spot, and the combined use with other therapies can improve the efficacy and expand the beneficiary population. Combination therapy can expand the indications and applicable population of immunotherapy and effectively improve drug efficacy. To date, the FDA has approved four PD-1/PD-L1 drug combination therapies. An increasing number of combined immunotherapies are expected to be approved in the future, and combined immunotherapies are becoming conventional treatments in the clinic.

Another challenge of tumor immunotherapy is the lack of biomarkers to predict the immunotherapy response. Tumor immunotherapy has achieved remarkable success. However, it is very important to screen groups that may benefit from treatment, predict drug efficacy, and guide clinical treatment. The selection of biomarkers has become the key factor for immunotherapy treatment. The detection of molecular biomarkers such as PD-L1, TMB, MSI, and gene mutation has been widely used, but it is well known that these biomarkers are not good enough to guide clinical decision making, and factors such as cancer type, tumor heterogeneity, and dynamic changes in tumors will affect the accuracy and specificity. Some patients who may benefit from treatment will be missed by these methods, while some patients with high PD-L1 expression and TMB-H are not sensitive to immunotherapy at all. Therefore, a better understanding of the mechanism of cancer immunotherapy, improving existing biomarkers, and developing new tumor markers are important future directions of immunotherapy. Studies have shown that the TME is also an important factor affecting the effect of immunotherapy. According to the degree of infiltration, tumors can be divided into different types. Generally, tumors with a high level of immune cell infiltration (“hot” tumors) are more likely to respond to immunotherapy, while “cold” tumors generally need to be transformed into hot tumors through treatment. New biomarker systems integrating the results of immune profiling, clinical history and tumor biology are being developed with the hope of predicting therapeutic outcomes before the application of immunotherapy.

Novel technologies are emerging to solve these clinical problems. New strategies, such as CRISPR-mediated screening and cell engineering, enable the identification and targeting of specific genes that sensitize tumor cells to immunotherapy or potentiate immune cells for sustained tumor-targeting capability. Neoantigen identification and TCR-T cells have also brought new hope for immunotherapy of certain types of cancer that are not sensitive to checkpoint inhibitors and CAR-T cells. Other novel technologies, such as TIL therapy, DC-based therapy and CAR-NK cell therapy, are also making ground-breaking progress, and products could be expected in clinical application very soon.

Overall, a combination of solid research on tumor immunology and advanced technology for manipulating immune cells will shed light on the future development of cancer immunotherapy. The advancement of cancer immunotherapy calls for more integrated clinical and basic research programs, which will then allow for the analyses of unmet clinical needs in a comprehensive manner and subsequent guidance of research directions.

## Supplementary information

Supplementary Information
