# Supplementary material for: Therapeutic targets and biomarkers of tumor immunotherapy: response versus non-response
Source: Signal Transduct Target Ther. 2022 Sep 19;7:331. doi: 10.1038/s41392-022-01136-2 (PMC9485144; doi:10.1038/s41392-022-01136-2)
Supplement: Supplementary file 1 — Supplementary Information [file 41392_2022_1136_MOESM1_ESM.pdf]

SPRINGER NATURE

Author Services

Editing Certificate

This document certifies that the manuscript

Therapeutic Targets and Biomarkers of Tumor Immunotherapy: Response versus No-response

prepared by the authors

Dong-rui Wang, Xian-lin Wu, Ying-li Sun

was edited for proper English language, grammar, punctuation, spelling, and overall style by one or more of the highly qualified native English speaking editors at SNAS.

This certificate was issued on **July 20, 2022** and may be verified on the SNAS website using the verification code **EE9D-242C-A3E7-9404-4D2P**.

Neither the research content nor the authors' intentions were altered in any way during the editing process. Documents receiving this certification should be English-ready for publication; however, the author has the ability to accept or reject our suggestions and changes. To verify the final SNAS edited version, please visit our verification page at secure.authorservices.springernature.com/certificate/verify.
If you have any questions or concerns about this edited document, please contact SNAS at support@as.springernature.com.

SNAS provides a range of editing, translation, and manuscript services for researchers and publishers around the world.
For more information about our company, services, and partner discounts, please visit authorservices.springernature.com.
